# Versatile and Robust Reservoir Computing with PWM‐Driven Heterogenous *R*–*C* Circuits

**DOI:** 10.1002/advs.202416413

**Published:** 2025-05-14

**Authors:** Zelin Ma, Huasen Yi, Ziping Zheng, Zhanyi Chen, Weicheng Liu, Yibing Chen, Bojun Cheng, Chang Cai, Shusheng Pan, Jun Ge

**Affiliations:** ^1^ School of Physics and Material Science Guangzhou University Guangzhou Higher Education Mega Center Panyu District Guangzhou 510006 China; ^2^ Research Center for Advanced Information Materials (CAIM) Huangpu Research & Graduate School of Guangzhou University Sino‐Singapore Guangzhou Knowledge City, Huangpu District Guangzhou 510555 China; ^3^ Microelectronics Thrust The Hong Kong University of Science and Technology (Guangzhou) No. 1 Duxue Road, Nansha District Guangzhou 511466 China; ^4^ Key Lab of Si‐based Information Materials & Devices and Integrated Circuits Design Department of Education of Guangdong Province Guangzhou Higher Education Mega Center, Panyu District Guangzhou 510006 China

**Keywords:** pulse width modulation, reservoir computing, resistor‐capacitor circuit, robustness, versatility

## Abstract

Physical reservoir computing (PRC) holds great promise for low‐latency, energy‐efficient information processing, yet current implementations often suffer from limited flexibility, adaptability, and environmental stability. Here, a PRC system based on pulse‐width modulation (PWM)‐encoded resistor‐capacitor (*R*–*C*) circuits is introduced, achieving exceptional versatility and robustness. By leveraging customizable nonlinearities and dynamic timescales, this system achieves state‐of‐the‐art performance across diverse tasks, including chaotic time‐series forecasting (NRMSE = 0.015 for Mackey‐Glass) and complex multiscale tasks (94% accuracy for multiclass heartbeat classification). Notably, the design reduces relative errors by 98.4% across different device batches and under temperature variations compared to memristor‐based reservoirs. These features position the approach as a scalable, adaptive, and energy‐efficient solution for edge computing in dynamic environments, paving the way for robust and practical analog computing systems.

## Graphical abstract

A robust physical reservoir computing (PRC) system using pulse‐width modulation (PWM)‐encoded *R*–*C* circuits is introduced. By enabling customizable nonlinearities and timescales, this system achieves state‐of‐the‐art accuracy in chaotic, multiscale, and multiclass tasks while maintaining stability across temperature variations and device batches. Its highly customizable design positions it as a valuable and practical solution for real‐time edge computing.

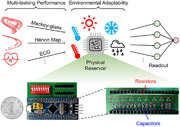

## 1 Introduction

The rapid pace of technological advancement increasingly relies on processing information with exceptional speed and precision.^[^ ^1^ ^]^ While deep neural networks (DNNs) have driven significant breakthroughs,^[^ ^2^, ^3^ ^]^ they typically require extensive training on large‐scale models. There is a growing demand for small, efficient models capable of quick inference and rapid adaptation, with reservoir computing (RC) standing out as a promising alternative.^[^ ^4^, ^5^ ^]^ RC models follow a three‐layer architecture: an input layer for receiving and preprocessing data, a reservoir layer where nonlinear recurrent dynamics are triggered by input signals, and an output layer that recombines signals to produce the final output. Unlike DNNs, where extensive parameter tuning is required, RC only trains the readout layer while keeping the reservoir's internal connections fixed. This drastically reduces the number of parameters and computational overhead.

In addition to software approaches, physical reservoir computing (PRC) directly utilizes inherent physical processes for computation, where an untrained physical ‘reservoir’ transforms inputs into the trainable output layer.^[^ ^6^, ^7^ ^]^ This approach thus, in theory, offers much higher computational efficiency compared to digital methods. PRC has been explored in various fields including analog circuits,^[^ ^8^, ^9^ ^]^ optics,^[^ ^10^ ^]^ spintronic nano‐oscillators,^[^ ^11^ ^]^ memristors,^[^ ^12^, ^13^, ^14^, ^15^, ^16^ ^]^ randomized networks^[^ ^17^, ^18^ ^]^ and small‐scale quantum computers.^[^ ^19^, ^20^ ^]^ Moreover, the sensing elements can be integrated with the computing hardware, making them particularly attractive for low‐latency applications. For example, a fully analog PRC system using memristors has been proposed for real‐time, power‐efficient signal processing, with analog pulse‐amplitude modulation (PAM) signals directly input to the physical reservoir. However, PRC's versatility remains limited due to the inherently fixed properties of physical systems. Although adaptive PRC has been proposed to address this issue by enabling systems to tune or expand their dynamics,^[^ ^21^, ^22^, ^23^, ^24^ ^]^ customized and precise control over system dynamics to meet task requirements remains an open challenge.

Another major challenge for PRC systems is the issue of reliability. In digital RC, the weights can be trained once and copied to millions of computers without worrying about hardware variations or environmental conditions. In contrast, the robustness of physical reservoirs is greatly restricted by their vulnerability to physical constraints. For example, physical reservoirs utilizing nonlinear semiconductor devices, such as transistors and diodes, are particularly susceptible to temperature‐induced issues, including leakage currents and threshold voltage shifts. This could affect the inference accuracy of the PRC system during deployment at varying temperatures. Moreover, emerging nanodevices like memristors, which are widely explored for their computational efficiency in RC,^[^ ^13^, ^25^, ^26^ ^]^ suffer from significant device‐to‐device variations, further complicating the replication of trained systems and hindering large‐scale deployment. Thus, developing robust PRC systems that maintain performance across varying environmental conditions and device batches is essential for their practical implementation.

**Figure 1:**
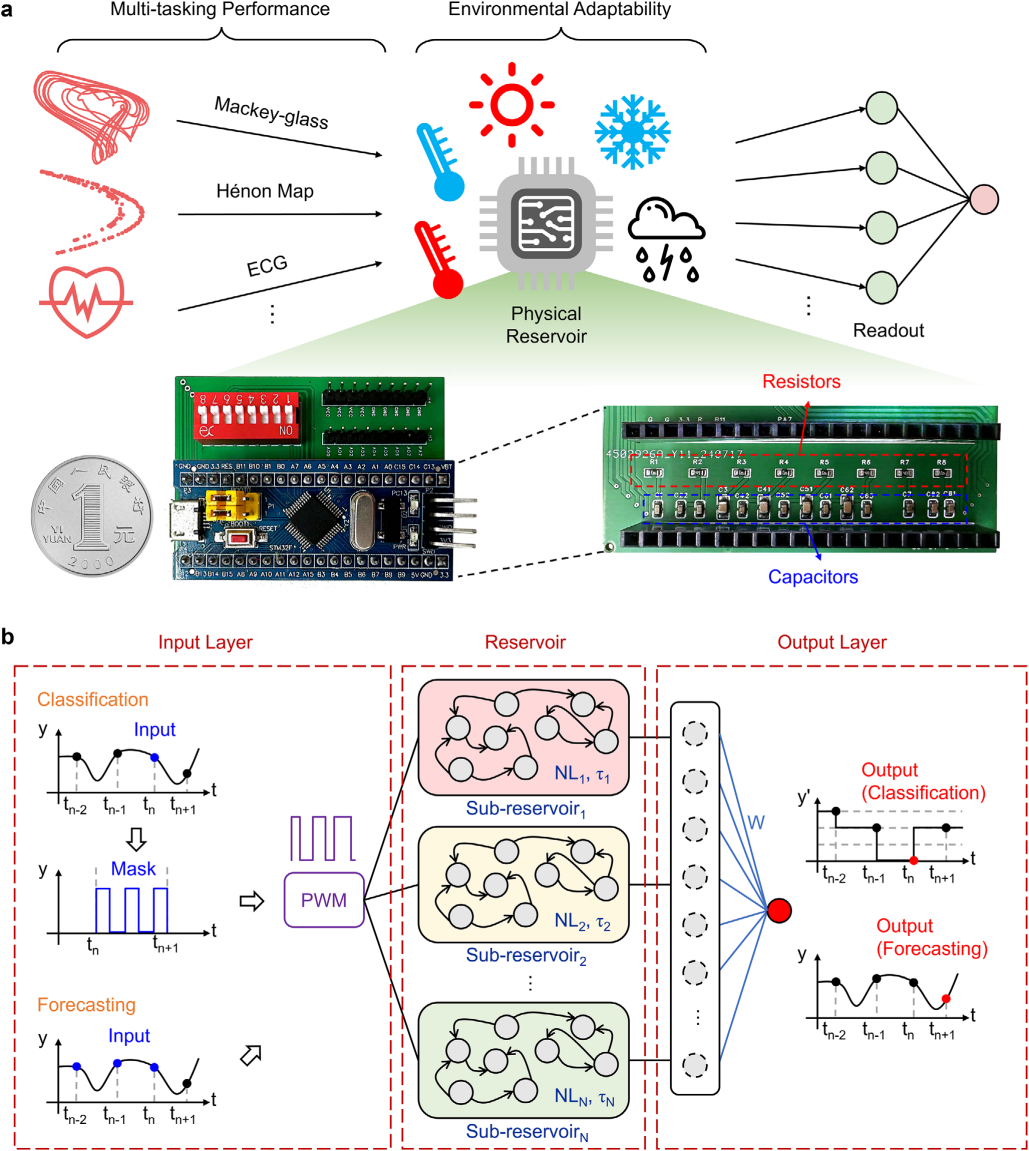
A PRC system based on grouped *R*–*C* circuits with PWM encoding method. a) Concept of versatile and robust physical reservoir and optical images of the hardware system. A coin is included for size reference. The system consists of a blue microcontroller board, mounted on a custom‐built green *R*–*C* circuits board to optimize the layout. The *R*–*C* board features eight series *R*–*C* circuits, functioning as a grouped reservoir, and provides eight output pins for measuring the output voltages across the capacitors. b) Schematic of the grouped reservoir used for time‐series classification and forecasting. The grouped reservoir comprises several sub‐reservoirs, each with different nonlinearity and diverse time constants, implemented using *R*–*C* circuits with varied parameters. Two types of node modes are utilized: virtual nodes (created through the masking process) and intermediate nodes (composed of reservoir outputs from the current and several previous time steps). These signals are first processed through the PWM module before being fed into the grouped reservoir. The reservoir states are then collected and fed into the software‐based readout layer. The readout weights are trained through linear regression or ridge regression.

In this work, we present a versatile and robust PRC system based on a set of resistor‐capacitor (*R*–*C*) circuits (**Figure** **1**a). Traditionally, *R*–*C* circuits are viewed as linear systems, which often limits their nonlinearity and state richness for RC tasks. Our approach addresses this limitation by encoding input data through voltage‐controlled pulse width modulation (PWM), a widely used, cost‐effective technique in control systems, which introduces the necessary nonlinearity to *R*–*C* circuits. Crucially, we show that by merely engineering the parameter of the resistor or capacitor in *R*–*C* circuits, both distributed nonlinearity (NL) and diverse temporal dynamics (τ) can be precisely obtained in a customizable way. We validate the flexibility of our approach by experimentally tackling three benchmark tasks: the NARMA2 system, Mackey‐Glass series, and Hénon map. Impressively, our system achieves state‐of‐the‐art accuracy on all tasks with a compact reservoir size of just 40 nodes, highlighting the high efficiency in feature extraction. We further demonstrate that this system can adapt with multiscale tasks requiring multiple timescales and nonlinearities, such as multiple‐speed Hénon map and multiclass arrhythmic heartbeat classification—areas where conventional physical reservoirs with homogeneous dynamic properties struggle. Notably, thanks to the minimalist and temperature‐resilient design of the *R*–*C* circuit, our system significantly outperforms simulated nonlinear analog circuits and memristor systems, maintaining stable performance even at 80 °C across different batches. Therefore, our PRC system offers a versatile, robust, and low‐cost solution for lightweight analog computing and real‐time temporal signal processing, paving the way for practical implementation in low‐power edge devices.

## 2 Results

### 2.1 Nonlinearity Introduction Through PWM Encoding in the *R*–*C* circuits

Traditional PRC systems often rely on PAM for input signal encoding, where information is conveyed by varying the amplitude of the input voltage. Here we encode the input signal with PWM to enhance the nonlinearity of the *R*–*C* reservoir (Figure 1b). For classification tasks, the input signal is pre‐processed by multiplying it with a mask matrix of 1 and ‐1 values, generating *N* virtual nodes.^[^ ^27^ ^]^ In prediction tasks, however, the input signal is processed directly: *N* intermediate nodes are created by combining the current output with the previous *N*−1 reservoir outputs, unless specified otherwise. A detailed explanation and summary of the node selection strategies can be found in Note S5 and Table S4 (Supporting Information). To implement PWM encoding, we leverage the built‐in PWM module within the microcontroller unit, converting the input into PWM signals. Each input signal then generates *M* (number of physical nodes) × *N* (number of virtual or intermediate nodes) reservoir states, which are fed into a software‐based linear or ridge regression network for readout (see more details in Experimental Section).

**Figure 2:**
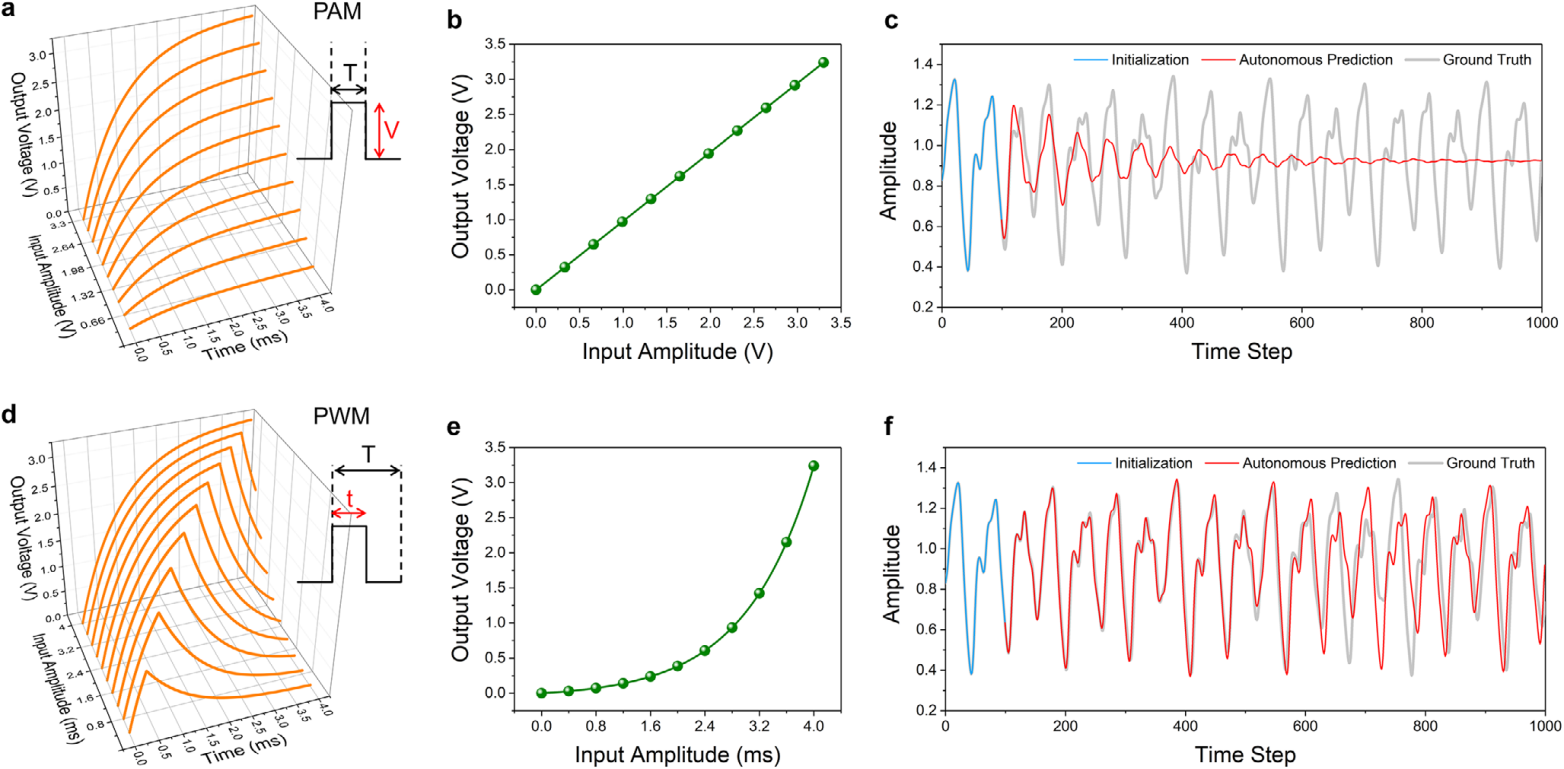
Comparison of PAM and PWM encoding in the *R*–*C* circuits reservoir. a, d) Output voltage of the *R*–*C* circuit with a time constant of 1 ms under PAM and PWM, respectively. Insets show the input pulse with a constant duration of *T* = 4 ms. For PAM, the input signal amplitude is encoded in the pulse voltage ranging from 0 to 3.3 V, while for PWM, it is encoded in the pulse width ranging from 0 to *T*. b, e) Relationship between input amplitude and output voltage for PAM and PWM, respectively, where output voltages are extracted from the final moment of *T* in (a) and (d). c, f) Autonomous prediction tests of the chaotic Mackey‐Glass time series under PAM and PWM.

To highlight the significance of PWM encoding, we start with a comparison of the response and performance of PAM and PWM through numerical simulations. **Figure** **2**a illustrates the input‐output relationship of the *R*–*C* reservoir using PAM. As an example, pulses with varying voltages (*V_in_*) ranging from 0 to 3.3 V, but with the same duration *T* (4 ms), are applied to a certain resistor (*R* = 40 kΩ)‐capacitor (*C* = 25 nF) circuit. The output voltage at the final moment of *T* can be expressed as:

(1) $$\begin{array}{c} V_{out}\;\left(T\right)=V_{in}\;\left(1-e^{-\frac{T}{\tau }}\right) \end{array}$$

where τ = *R* × *C* = 1 ms is the time constant of the *R*–*C* circuit. Apparently, PAM leads to linear relationship between *V_in_* and *V_out_* (*T*) (Figure 2b). Figure 2c shows the system's autonomous prediction of Mackey‐Glass time series with the ground truth (gray line) over time. In this case, the number of intermediate nodes was set to 30. After a 1000‐time‐step training process (Figure S1, Supporting Information), a 100‐time‐step initialization phase (azure line) is necessary to set up the reservoir's internal states, as chaotic systems are highly sensitive to initial conditions. The linear reservoir encoded by the PAM fails to replicate the chaotic system's dynamics during the autonomous prediction, causing the forecast to converge to a stable point (red line). In contrast, Figure 2d shows the same *R*–*C* reservoir's response when using PWM encoding. Specifically, the input is encoded as the pulse width *t*, which ranges from 0 to *T* (the fixed period of the PWM signal), while the pulse voltage remains constant at *V_const_* (3.3 V). This time‐domain encoding is a core characteristic of PWM, where the input value is represented by the width of pulses within a fixed time period. In this scenario, the output can be expressed as:

(2) $$\begin{array}{c} V_{out}\;\left(T\right)=V_{const}\;\left(1-e^{-\frac{t}{\tau }}\right)e^{-\frac{T-t}{\tau }} \end{array}$$

where *t* is the time duration of the pulse. Figure 2e indicates that the nonlinear characteristic between *t* and *V_out_* (*T*) is imparted by the PWM. Therefore, as shown in Figure 2f, the *R*–*C* circuit reservoir with PWM successfully and autonomously predicts chaotic Mackey‐Glass time series up to ≈500 time steps (from 100 to 600). This highlights the crucial role that PWM plays in introducing nonlinearity into the system, allowing the reservoir to capture complex temporal dynamics that the linear encoding of PAM fails to address.

### 2.2 Performance of the PRC System Across Different Types of Benchmark Tasks

**Figure 3:**
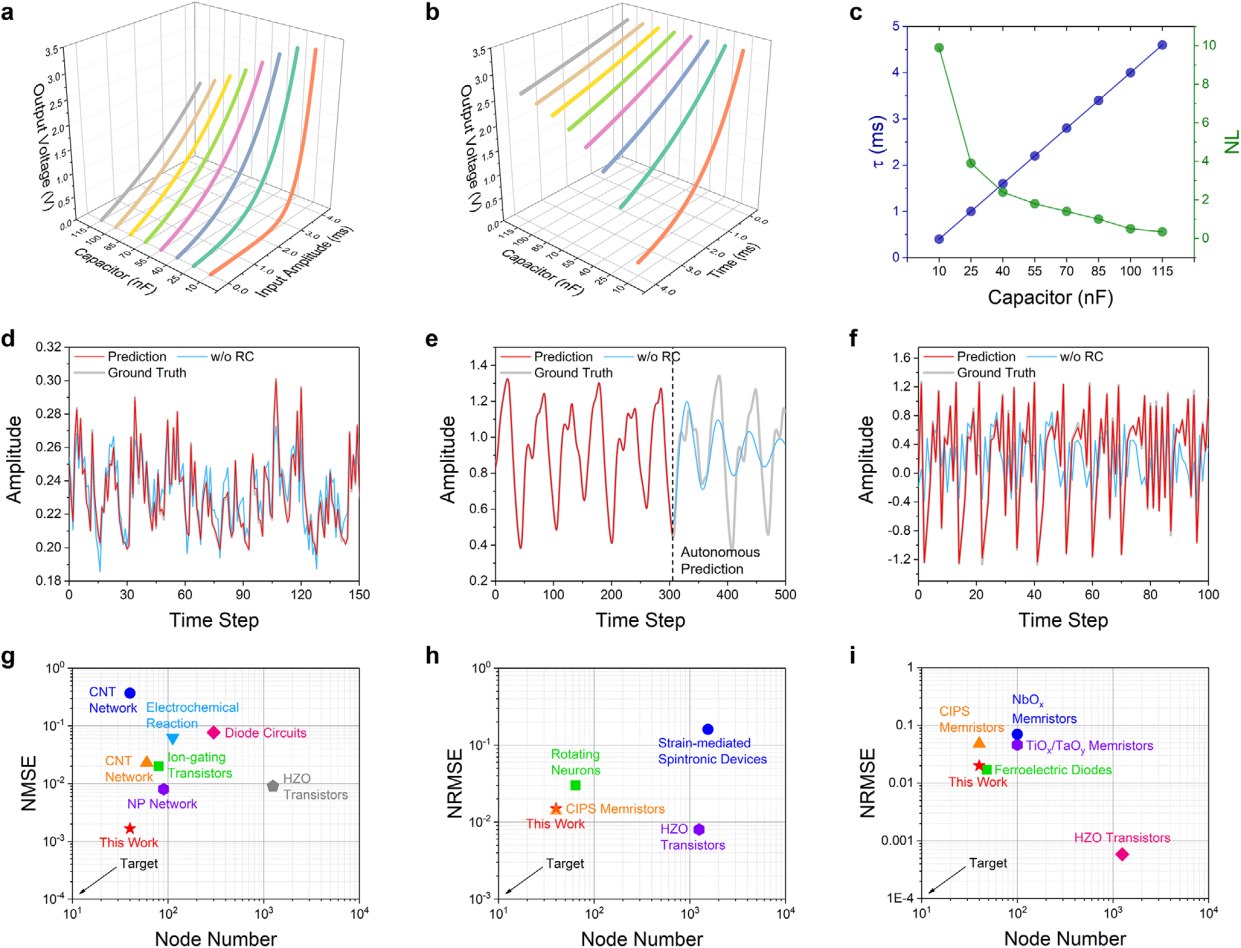
Comprehensive analysis of nonlinearity and temporal dynamics in grouped *R*–*C* circuits reservoir. a) Input‐output relationships of eight *R*–*C* circuits with capacitances from 10 to 115 nF. b) Relaxation process of eight *R*–*C* circuits after being fully charged to 3.3 V. c) Relationship between the time constant, nonlinearity factor, and capacitance in the grouped reservoir. d‐f) Prediction results for NARMA2, Mackey‐Glass, and Hénon map time series. Gray, red, and blue lines represent the ideal target, reservoir output, and output without the reservoir, respectively. g‐i) Prediction errors of the NARMA2, Mackey‐Glass, and Hénon map tasks, along with the total node numbers, are compared to other state‐of‐the‐art physical reservoirs including carbon nanotubes (CNT) network,^[^ ^28^, ^29^ ^]^ nanoparticles (NP) network,^[^ ^18^ ^]^ electrochemical reaction,^[^ ^30^ ^]^ ion‐gating transistors,^[^ ^14^ ^]^ Hf_0.5_Zr_0.5_O_2_ (HZO) transistors,^[^ ^31^ ^]^ strain‐mediated spintronic devices,^[^ ^32^ ^]^ ferroelectric diodes,^[^ ^15^ ^]^ NbO_x_ memristors,^[^ ^33^ ^]^ TiO_x_/TaO_y_ memristors,^[^ ^27^ ^]^ CuInP_2_S_6_ (CIPS) memristors,^[^ ^34^ ^]^ diode circuits^[^ ^35^ ^]^ and rotating neurons.^[^ ^9^ ^]^

Using different combinations of capacitors and resistors, we can precisely achieve distributed nonlinearities and diverse temporal dynamics. To simplify the process and enable easier comparison, we fixed the resistance at 40 kΩ and varied the capacitance between 10 and 115 nF. **Figure** **3**a shows the input‐output relationship of our eight *R*–*C* circuits under PWM encoding, demonstrating that as the capacitance increases over an 11‐fold range, the circuit's nonlinearity changes significantly. The nonlinearity factor (NL) was used to quantify this (see the “Experimental Section” section for calculation details), with a higher NL indicating a greater degree of nonlinearity. As indicated in Figure 3c (green line), the NL can be continuously adjusted from 0.3 to 9.9, covering a range from predominantly linear to highly nonlinear behavior. Figure 3b further demonstrates the fading memory of the eight *R*–*C* circuits after being fully charged to 3.3 V, with higher capacitance leading to an extended memory effect. The time constant (τ), which quantifies the circuit's fading memory, ranges from 400 µs to 4.6 ms (blue line in Figure 3c). With the parameter selection, this precise control over both nonlinearity and temporal dynamics across an arbitrary range represents a significant advancement in the development of more powerful, versatile, and adaptable computing systems.

We begin by assessing the versatility of our system through a series of benchmark tasks, including NARMA2, Mackey‐Glass, and Hénon map time series predictions. These tasks are commonly used to evaluate the performance of RC systems and they each demand varying dynamics (see more discussion in Note S1 and Table S1, Supporting Information). Due to these differing requirements, few PRC systems are capable of handling all three tasks effectively and simultaneously. Here, we experimentally conducted these three benchmark tasks using the same reservoir configuration based on *R*–*C* circuits. The number of intermediate nodes was set to 5 and we defined the input data interval as *T* = 4 ms. The reservoir consisted of 40 nodes, generated from 5 intermediate nodes across 8 physical circuits. These states were then applied to the readout layer, a 41 × 1 network that included an additional bias term, to produce the prediction for the next time step. The prediction outcomes are illustrated in Figure 3d–f. In each subplot, the red curves represent the predictions from our PRC system, the gray curves indicate the ground truth, and the blue curves show predictions without the use of reservoir. Due to technical limitations, autonomous predictions for the Mackey‐Glass and Hénon map tasks were not conducted experimentally; instead, the results were obtained through simulations (Figures S2 and S3, Supporting Information). Our grouped reservoir consistently produces predictions that closely align with the ground truth across all tasks, markedly outperforming the non‐reservoir predictions. It is particularly noteworthy that, while the non‐reservoir system performs well in single‐point prediction for the Mackey‐Glass task, it fails to sustain autonomous prediction, as illustrated in Figure 3e. This occurs because, in autonomous prediction, the system feeds its own prior predictions as inputs, but lacks the internal memory and dynamic complexity to capture the long‐term dependencies of chaotic time series. Consequently, it quickly loses accuracy and converges toward a stable output as the error compounds over time. In contrast, the reservoir system successfully maintains autonomous prediction, effectively capturing complex temporal dynamics and preserving accuracy over extended time steps, as shown in Figure S2 (Supporting Information). Quantitatively, the normalized mean squared error (NMSE) for the NARMA2 task was recorded at 0.002, while the normalized root mean squared errors (NRMSE) for the Mackey‐Glass and Hénon map prediction tasks were 0.015 and 0.021, respectively.

To highlight the strong performance of our reservoir, we present a comparative analysis in Figure 3g–i, contrasting our results with those from other state‐of‐the‐art PRC systems. It is worth noting that most other PRC systems report results for only two or even one of these tasks, while our system successfully tackles all three. Despite utilizing only 40 nodes, our reservoir consistently delivers accuracy that ranks among the best across all tasks (for detailed accuracy values, see Table S2, Supporting Information). This demonstrates not only the versatility of our approach in handling diverse prediction challenges, but also its efficiency in achieving high performance with minimal computational resources. However, we recognize that the current PCB‐based realization of the *R*–*C* circuit is not optimal in terms of footprint, especially when compared to the nanoscale implementations referenced in Figure 3g–i. This relatively bulky form factor stems from the use of discrete components and off‐chip interconnections, which inherently limit integration density. Thus, further miniaturization is essential for broader applicability.

### 2.3 Performance of the PRC System for Multiscale Tasks

**Figure 4:**
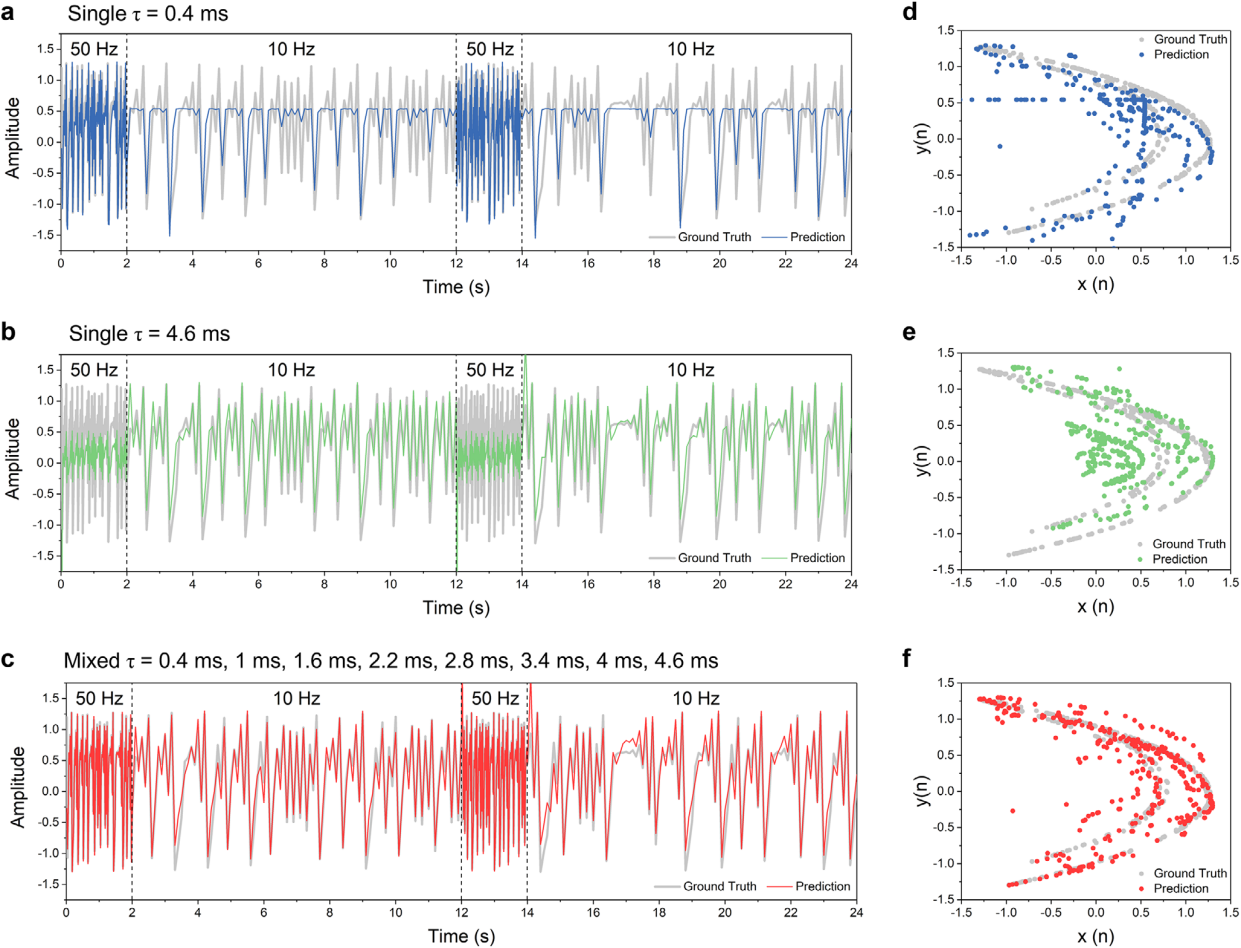
Frequency adaptability of the grouped *R*–*C* circuits reservoir. a‐c) Predicted Hénon map time series results for two single time constant reservoirs and the grouped reservoir. The modified Hénon map time series includes two different frequencies, 10 Hz and 50 Hz. Each segment with a different frequency consists of 100 time steps. The predicted result for the parallel reservoir consisting of a time constant of 400 µs is shown in (a), while the result with a time constant of 4.6 ms is shown in (b). The predicted result for the grouped reservoir with a wide range of time constants from 400 µs to 4.6 ms, is presented in (c). d‐f) 2‐D display of the predicted results of the corresponding reservoirs.

Real‐world applications, such as predicting motion trajectories, often require the ability to handle a wide range of speed conditions, which in turn demands high adaptability from the PRC system. To simulate such a scenario, we modified the traditional Hénon map prediction task by incorporating signals with varying frequencies, ranging from 10 to 50 Hz—a fivefold variation. This variation is designed to test the reservoir's capability to manage diverse temporal dynamics. A reservoir with a single time constant is typically inadequate for such tasks. For example, in our experiments, a reservoir consisting of eight parallel, identical sub‐reservoirs, each with a τ of 400 µs and distinct mask sequences, performed well in predicting the 50 Hz portion of the signals. However, this setup struggled significantly with the 10 Hz portion, as shown in **Figure** **4**a,d. This illustrates the limitation of using a reservoir with a narrow time constant range—effective at high frequencies but insufficient for slower dynamics. In contrast, a different configuration using a parallel reservoir with a much longer τ of 4.6 ms performed well in predicting the 10 Hz portion of the signals, as shown in Figure 4b,e. However, this setup struggled significantly with the 50 Hz portion, again highlighting the limitations of a reservoir with fixed temporal dynamics.

Our optimized reservoir offers a wide range of time constants, allowing it to handle multiple timescales simultaneously. As shown in Figure 4c,f, this configuration enabled the system to accurately predict both the 10 and 50 Hz portions of the signals within the same framework, without the need for feedback control. This adaptability can be further improved by adjusting the τ distribution to cover an even broader range, making the system more capable of managing tasks involving complex and varying temporal dynamics.

**Figure 5:**
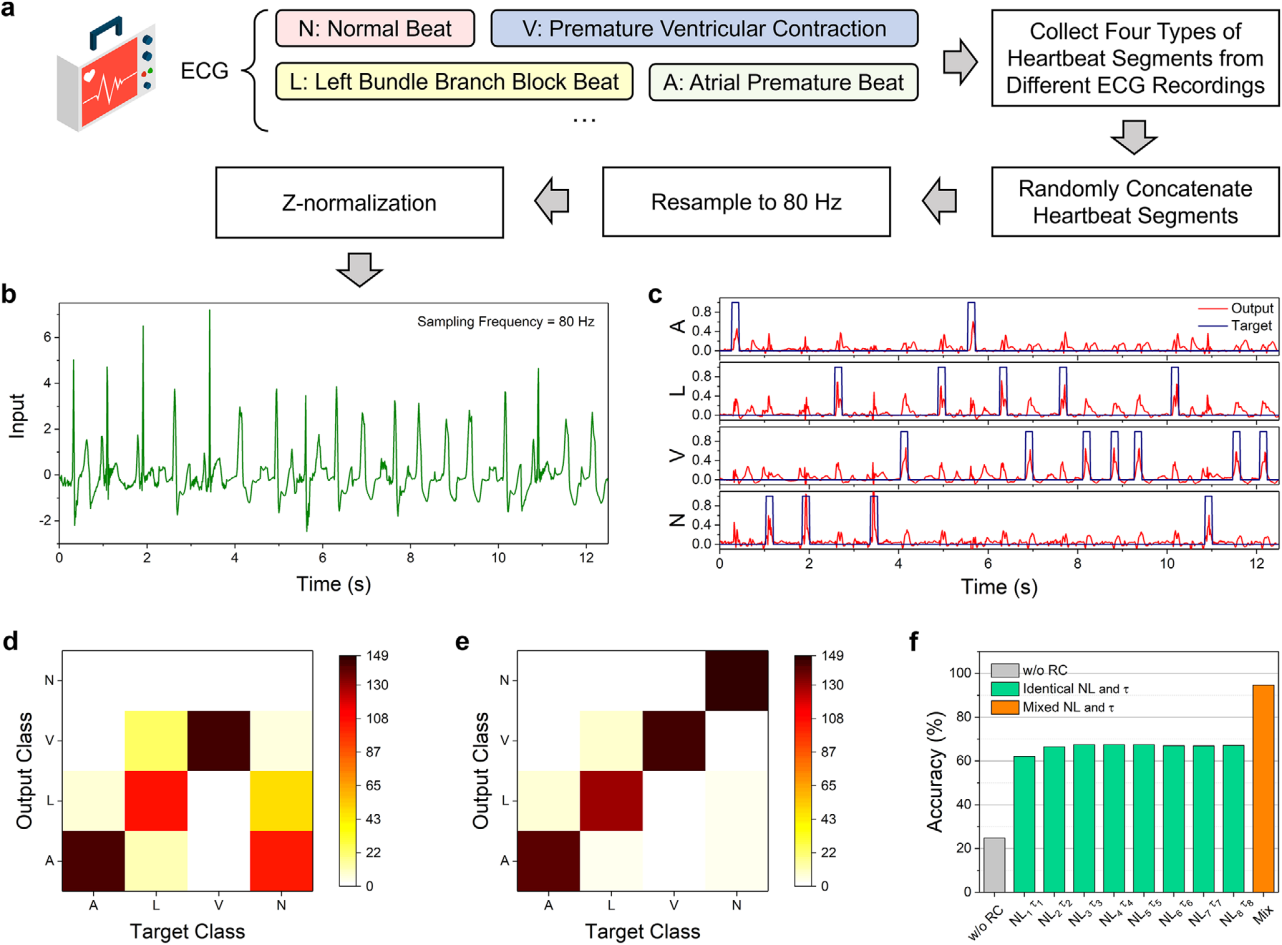
Multiclass arrhythmic heartbeat classification using the grouped *R*–*C* circuits reservoir. a) Four main classes of the MIT‐BIH dataset and the preprocessing process of heartbeat input signals. b) A segment of heartbeat signals randomly combined from four different heartbeat categories. The first 80% of the whole heartbeat signals is used for training, with the remainder reserved for testing. c) Target signals and output signals of the grouped reservoir system. The classification accuracy is determined by the highest output signal peak within the set time window. d) Confusion matrix showing the recognition results of a traditional parallel reservoir consisting of eight identical *R*–*C* circuits. e) Confusion matrix of the recognition results of the grouped reservoir consisting of eight different *R*–*C* circuits, with time constants and nonlinearity factors covering all values shown in Figure 3c. f) Comparison of recognition accuracy between parallel reservoirs with identical time constants and nonlinearity factors, and grouped reservoir with mixed time constants and nonlinearity factors.

To further demonstrate the capabilities of our system to handle complex time‐series tasks, we carried out the multiclass arrhythmic heartbeat detection task using the MIT‐BIH heart arrhythmia database.^[^ ^36^ ^]^ As shown in **Figure** **5**a, the MIT‐BIH dataset includes electrocardiogram (ECG) signals from 48 subjects, with our focus on four primary groups: A, L, V, and N. We began by selecting 750 heartbeat data segments from each of the four categories and randomly concatenated them to form a long electrocardiogram waveform sequence of 3000 heartbeat segments. This sequence was then re‐sampled at a frequency of 80 Hz (input data interval = 12.5 ms), and the amplitude was normalized using Z‐normalization (Figure 5b). For this classification task, the number of virtual node was set to 5, resulting in a mask data interval of *T* = 12.5 ms / 5 = 2.5 ms. The reservoir states from 40 nodes were fed into the readout layer (a 41 × 4 network) to generate classification data at each time step. A classification was considered correct if the output of the corresponding neuron in the readout layer reached its maximum value within the set time window.

A portion of the four output and target signals of the testing process are shown in Figure 5c. Evidently, the output signal can effectively fit the target signal and generates the highest peak when a corresponding heartbeat occurs. An impressive accuracy of 94% is achieved for this four‐class classification (Figure 5e), significantly outperforming an electrochemical network reservoir with a delay feedback line (accuracy = 88%).^[^ ^37^ ^]^ In comparison, when the input signal is directly fed into a linear regression network, the recognition accuracy is only 25% (Figure 5f), which is equivalent to random guessing given that there are four categories. This highlights the importance of the reservoir in extracting useful features from the data.

Additionally, we tested traditional parallel reservoir systems with eight identical sub‐reservoirs, each applying a distinct mask sequence, but with only single nonlinearity and temporal characteristics. Despite simulating with various reservoir parameter configurations, all accuracies remained below 67% (Figure 5d,f). This lower accuracy can be attributed to the diversity of arrhythmias, as each type has unique and subtle ECG signal characteristics. Systems with limited feature extraction capabilities struggle to capture these differences effectively. In contrast, our grouped reservoir harnesses a wider range of nonlinear and temporal features, allowing for more accurate processing of complex temporal signals.

### 2.4 Comparative Robustness of the PRC System Under Temperature and Assembly Variability

**Figure 6:**
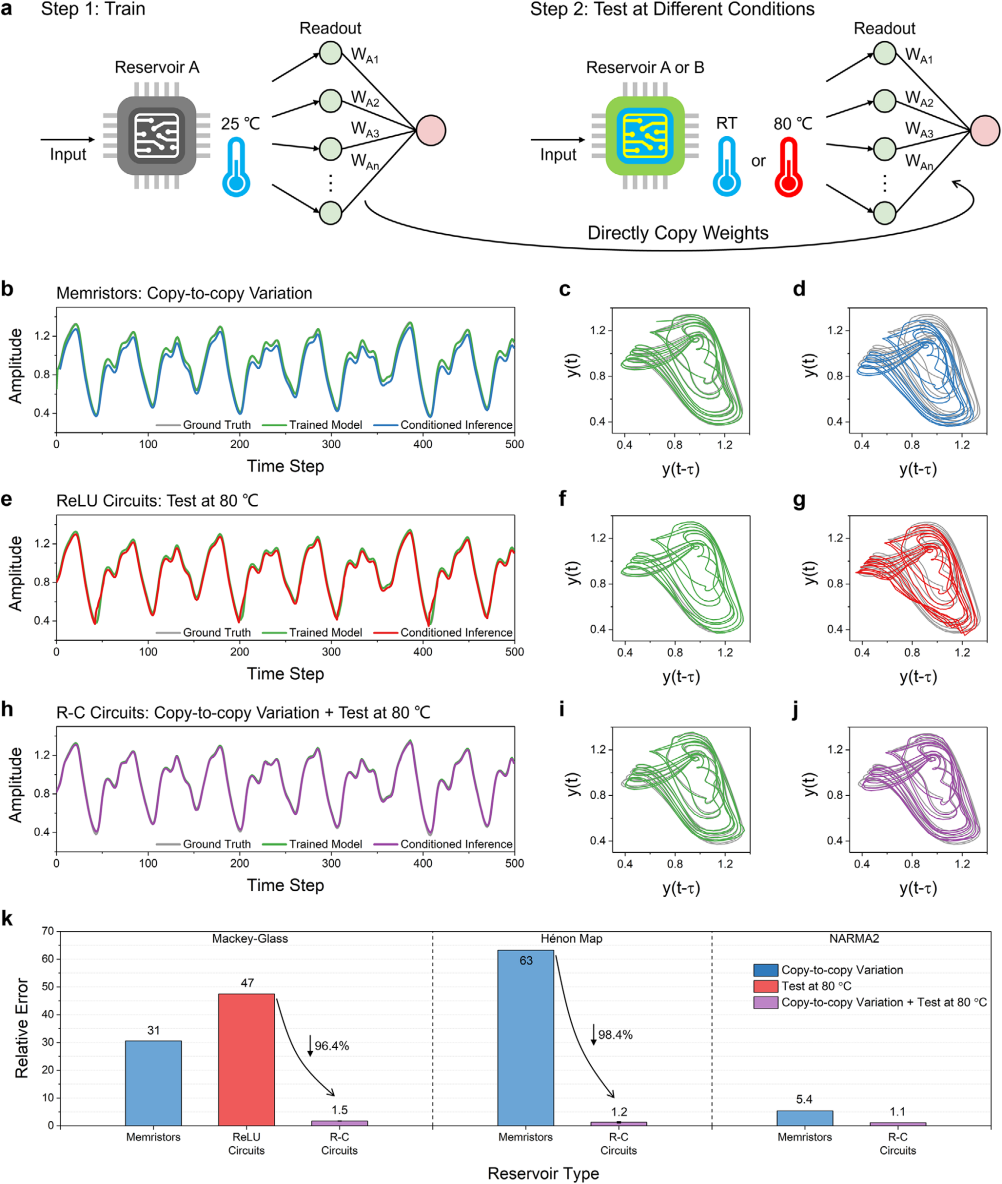
Copy‐to‐copy variability and temperature sensitivity of the grouped *R*–*C* circuits reservoir. a) Steps for robustness testing. b) Prediction results of the memristors reservoir A (green curve) and B (blue curve) at RT for the Mackey‐Glass time series. c‐d) Phase space of the predicted results in (b). e) Prediction results of the ReLU circuits reservoir A at RT (green curve) and A at 80 °C (red curve) for the Mackey‐Glass time series. f,g) Phase space of the predicted results in (e). h) Prediction results of the *R*–*C* circuits reservoir A at RT (green curve) and B at 80 °C (purple curve) for the Mackey‐Glass time series. i,j) Phase space of the predicted results in (h). k) Comparison of relative test errors across different types of reservoirs including *R*–*C* circuits, memristors and ReLU circuits for the NARMA2, Mackey‐Glass and Hénon map time series.

Minimizing the differences between physical reservoirs is essential to reduce training costs. If these differences are substantial, each PRC system would require retraining of the readout layer weights. Similarly, influences of external environments can cause instability and drift in the reservoir, rendering pre‐trained weights ineffective. **Figure** **6**a illustrates the PRC inference process. The trained weights in the readout layer, obtained from a physical reservoir A at room temperature (RT), are directly applied in the inference stage which can be performed by a replicated reservoir A (denoted as B), by reservoir A at elevated temperatures, or by reservoir B at elevated temperatures. Specifically, we evaluated the inference performance of memristors reservoir and ReLU circuits reservoir through simulations (see Notes S2 and S3, Supporting Information for simulation details). For the memristors reservoir, we examined the effect of copy‐to‐copy variations of reservoir B at RT, setting the variation coefficient (C_v_) to 5%, which reflects the current state‐of‐the‐art for memristor technology.^[^ ^38^, ^39^, ^40^ ^]^ For the ReLU circuits reservoir, we focused solely on the high‐temperature effects of reservoir A at 80 °C. Note that other physical imperfections such as read noise, low numerical precision and stochasticity are not considered in simulations. Figure 6b–d show the results of the memristors reservoir for the Mackey‐Glass one‐step prediction task. Although the trained model (green curve) closely aligns with the ground truth (grey curve), the inference results (blue curve) deviate significantly from the ground truth due to copy‐to‐copy variations. Similarly, the inference results of the ReLU circuits reservoir at 80 °C (red curve) also diverge from the ground truth (Figure 6e–g), attributed to the altered input‐output relationship of the ReLU circuits (see Figure S5, Supporting Information).

For comparison, we examined both effects of device variations and temperature drifts using two *R*–*C* circuits reservoirs which were fabricated with identical parameters (Figure S6, Supporting Information). To maximize copy‐to‐copy variability, one circuit was assembled using machine soldering for training process, while the other was assembled manually using hand‐soldering techniques for inference process. Figure 6h–j demonstrate the inference result of our replicated system at 80 °C (purple curve), indicating a close alignment with the trained model and the ground truth (please refer to Figure S7 (Supporting Information) for the testing setup and Figures S13–S15 (Supporting Information) for the raw voltage outputs of the *R*–*C* circuits). It's worth noting that our experiments account for unavoidable physical factors such as read noise and precision limitations, which are omitted in simulations. To quantitatively compare the inference performance of different types of reservoirs, the relative prediction error is defined as the ratio of the conditioned inference error (NMSE) to the inference error (NMSE) of the original trained model, as shown below.

(3) $$\begin{array}{c} Relative\;Error=\frac{Conditioned\;Inference\;Error\;\left(NMSE\right)}{Inference\;Error\;of\;Trained\;Model\;\left(NMSE\right)\;} \end{array}$$

Ideally, the relative error should be close to 1. Figure 6k displays the relative errors of various reservoirs across three benchmark tasks (see absolute errors in Figures S8–S10, Supporting Information). Due to device variations and temperature‐induced parameter drifts, the memristor and ReLU circuit reservoirs exhibit substantially higher relative errors, reaching 63 and 47 for the Hénon map and Mackey‐Glass tasks, respectively. In contrast, the *R*–*C* circuits reservoir exhibits relative errors close to 1 across all tasks, with a slight increase to ≈1.5 for the Mackey‐Glass task. This represents a 98.4% (Hénon map task) and 96.4% (Mackey‐Glass task) reduction in relative error compared to the memristors and ReLU circuits reservoirs, respectively. These results clearly highlight the superior robustness of the *R*–*C* circuits reservoirs against device variations and temperature fluctuations relative to other reservoir types.

## 3 Discussion

Our study demonstrates that PWM encoding can be effectively integrated into *R*–*C* circuit‐based PRC systems. By leveraging PWM, we introduce essential nonlinearity, enabling the reservoir to better capture complex temporal dynamics, such as chaotic series, that linear encodings like PAM cannot replicate. Moreover, by modulating pulse width rather than amplitude, distributed nonlinearities can be achieved alongside changes in the *R*–*C* circuit's time constant, resulting in a richer dynamic response that enhances classification and prediction performance. It is important to note that this finding is specific to *R*–*C* circuit‐based reservoirs, which exhibit inherently linear dynamics. For other reservoir systems with intrinsic nonlinearity, PAM may still be a viable encoding method, and its performance could vary depending on the system's characteristics. In addition to its superior capability in handling complex dynamics, PWM also offers advantages in terms of power consumption when compared to PAM.^[^ ^41^, ^42^ ^]^ Unlike PAM, which relies on continuous amplitude modulation, PWM minimizes power losses by switching between fully on and off states, avoiding the inefficiencies of continuously varying analog signals. Furthermore, PWM's superior noise immunity reduces the need for power‐intensive signal corrections. These advantages make PWM more energy‐efficient, especially in power‐sensitive and high‐efficiency applications.

A key advantage of our PRC system is its ability to precisely tailor both nonlinearities and time constants to meet the demands of diverse computational tasks. In this study, the nonlinearity variation reaches up to 3300%, while the time constant variation spans 1200%. These ranges can be further extended by adjusting the resistor or capacitor values. In contrast, emerging nanodevices often face significant challenges in controlling their nonlinearities and timescales. Although memristor system with random device‐to‐device variations have been suggested, the tunable range is uncontrollable. On the other hand, the nonlinearity of ReLU circuits can be modified by adjusting the voltage‐current characteristics of diodes, but the adjustable range of the diode's threshold voltage is very constrained. Other solutions include adjusting the input scheme by varying the input range and rate fed into the reservoir. However, modifying the input range and rate requires additional circuitry, which can introduce latency and increase the system's overall power consumption.

Recent studies have extensively explored emerging electronics for PRC due to their energy efficiency.^[^ ^7^ ^]^ In this work, each input is a pulse with a 3.3 V amplitude (equal to the MCU output voltage) and an average duration of 2 ms. Consequently, the maximum energy consumption per input is ≈(3.3 V)^2^ / 40 kΩ × 2 ms = 545 nJ. While this is relatively high compared to state‐of‐the‐art technologies like memristors (which can operate in the range of a few pJ^[^ ^26^ ^]^), it can be significantly reduced with straightforward adjustments. One approach is to increase the resistor value, which would require a smaller capacitor to be used to maintain the same time constant. Moreover, the *R*–*C* circuit has no strict operating voltage limitations, enabling further energy reduction by lowering the operating voltage. On the other hand, the relatively large area overhead of the current PCB‐level implementation of *R*–*C* circuits can be reduced by integrating them into CMOS‐compatible SoC platforms, where monolithic implementation on silicon is feasible. For instance, MOS capacitors, MOS resistors, and MOSFET‐based variable resistors can achieve nanoscale feature sizes, while benefiting from mature fabrication processes and well‐established design tool support. Table S3 (Supporting Information) presents a general comparison of physical reservoirs using different technologies across several evaluation metrics. Our proposed scheme offers unique advantages, including customization, copy‐to‐copy stability, and environmental robustness.

Regarding the distributed states of the reservoir, our results suggest that a wide range of nonlinearities and time constants not only enhances the RC system's versatility but also improves its performance on complex tasks. However, there is a trade‐off to consider. While a broader range in general offers the RC system more capability, it also increases the reservoir size and the complexity of the readout layer. In practice, the optimal range of distributed states depends on the specific application and its requirements. For tasks with multiple or variable temporal dynamics, a wider range is indeed advantageous. Conversely, for tasks with more consistent signal patterns, a narrower, well‐targeted range may yield better overall performance with lower computational overhead.

One of the key challenges for the proposed system is replacing the current software‐based readout layer with a fully hardware implementation capable of autonomous learning. Our *R*–*C* circuit reservoir is particularly well‐suited for this transition, as it produces voltage‐domain outputs that can be directly interfaced with a memristive memory array‐based readout circuit,^[^ ^9^, ^13^, ^15^ ^]^ leveraging Kirchhoff's law for analog vector‐matrix multiplication. To ensure signal integrity and prevent load‐induced variations, a buffer stage—such as a voltage follower—can be employed to decouple the reservoir from the readout layer and maintain stable operation. Our work also opens up interesting avenues for further PRC development. For example, while this work has focused on the robustness of our *R*–*C* circuit system to external conditions, our system also holds promise for treating external stimuli as assets for the PRC system (i.e., in‐sensor RC^[^ ^43^ ^]^). This could be easily achieved by replacing the environment‐insensitive resistors with stimulus‐sensitive resistors, such as photoresistors, thermistors, gas‐sensitive resistors, or even their parallel combinations for multiple modalities (Note S4, Supporting Information). This would enable the PRC system to fully perceive the external world in a highly fused manner, providing additional insights and situational awareness for specific tasks or challenges, thereby enhancing performance and bringing it closer to human‐like intelligence.

## 4 Experimental Section

### Design and Measurement of the Reservoir Hardware

The circuit schematic of the proposed system is shown in Figure S12a (Supporting Information). The entire test setup consists of three main components: a host personal computer (PC), an STM32 microcontroller unit (MCU) development board, and a custom‐designed PCB with 8 parallel *R*–*C* series circuits. The system operation procedure is illustrated in Figure S12b (Supporting Information). The PC functions as the host controller, responsible for data loading, parameter configuration, and user interaction. The MCU acts as the lower‐level controller, receiving commands from the PC and generating PWM control signals using its internal timer. These PWM signals were simultaneously applied to all eight *R*–*C* branches via a single general‐purpose input/output (GPIO) port. Since the PWM was generated directly in hardware, no digital‐to‐analog converter (DAC) was required, and the output voltage remains fixed at 3.3 V, the supply voltage of the MCU. For the 8 parallel *R*–*C* circuits, the resistance was fixed at 40 kΩ, and the capacitance values were 10, 25, 40, 55, 70, 85, 100, and 115 nF, respectively. The input voltage signal was applied to the resistor end of the *R*–*C* circuit, while the capacitor end was grounded. The voltage outputs were measured at the junction between the resistors and capacitors. The output voltages from the *R*–*C* circuits were measured either by a Keithley B2902A source measure unit (SMU) for full waveform acquisition, or by the MCU's built‐in 12‐bit analog‐to‐digital converter (ADC) for sampling of reservoir states. The resulting waveforms or sampled states were then transmitted back to the PC for further analysis and for training/testing of the readout layer.

### NARMA2 Prediction

NARMA2 was a challenging time series prediction benchmark commonly used to quantify the computational capabilities of brain‐like information processing systems. The target *y*(*k* + 1) was obtained from following equation:

(4) $$\begin{array}{c} y\;\left(k+1\right)=0.4y\left(k\right)+0.4y\left(k\right)y\left(k-1\right)+0.6u^{3}\left(k\right)+0.1 \end{array}$$

where *u*(*k*) = [0, 0.5] is a discrete white noise signal. The output *y*(*k* + 1) not only depends on the current input *u*(*k*), but was also related to the cross term of past two outputs *y*(*k*) and *y*(*k* −1). In typical applications, the relationship between *y*(*k* + 1) and *u*(*k*) was implicit and hidden, which makes the prediction difficult to solve. The training and testing phases for the NARMA2 task were both set to 600 time steps.

### Mackey‐Glass Prediction

Mackey‐Glass chaotic system has been used in various reservoir computing studies as a benchmark task. The Mackey‐Glass system was defined by

(5) $$\begin{array}{c} \;\frac{dy}{dt}=\beta \frac{y\left(t-\tau \right)}{1+y{\left(t-\tau \right)}^{n}}-\gamma y\left(t\right) \end{array}$$

where the system parameters γ, β, and *n* were set to the widely used values 0.1, 0.2, and 10, respectively. Additionally, the system was chaotic when τ > 16.8, and predictions become correspondingly more difficult. In this experiment, it set τ = 17 and the initial value *y*(0) = 1.2 following previous works. The objective of this task was for the system to output the signal at the next time step *y*(*k* + 1) when provided with the current signal *y*(*k*). The training and testing phases for the Mackey‐Glass task were both set to 1000 time steps. In the case of autonomous prediction, the 1000 testing time steps include 100 initial time steps for initialization.

### Hénon Map Prediction

Hénon map has been established as a typical discrete‐time dynamic system with chaotic behavior. It describes a nonlinear 2D mapping that transforms a point (*x*(*k*),  *y*(*k*)) on the plane into a new point (*x*(*k*  +  1),  *y*(*k*  +  1)), defined as follows:

(6) $$\begin{array}{c} x\;\left(k+1\right)=1+y\left(k\right)-1.4x^{2}\left(k\right) \end{array}$$

(7) $$\begin{array}{c} y\;\left(k+1\right)=0.3x\left(k\right)+w\left(k\right) \end{array}$$

where *w*(*k*) is a Gaussian noise with a mean value of 0 and a standard deviation of 0.05. The system can be described as an equation containing only *x* if it combine Equations (6) and (7). That is, an input *x*(*k*) value is applied to the reservoir system to predict the data for the next time step *x*(*k* + 1). The training and testing phases for the Hénon map task were both set to 400 time steps. In the case of autonomous prediction, the 400 testing time steps include 50 initial time steps for initialization. For the frequency adaptability test, the time steps involving two frequencies were also set to 400 for both the training and testing phases.

### Readout Function Training

A supervised learning algorithm was used to train the readout function in a digit mode by using Python. Linear or ridge regression method was used to calculate *W_out_*. It generated a target matrix *Y_target_* by combining the target vectors at all the time steps used for training and collected a state matrix *X* by combining the response vectors at all of the time steps used for training. Subsequently, the weight matrix *W_out_* was directly given by *W_out_* = *Y_target_* *X^T^* (*XX^T^*)† for linear regression, or *W_out_* = *Y_target_* *X^T^* (*XX^T^* + λ*I*)^−1^ for ridge regression, where the symbol † represents Moore‐Penrose pseudo‐inverse, λ is the regularization parameter and *I* is the identity matrix. In all the tasks mentioned in the article, ridge regression was used for the robustness test of RC systems, while linear regression was used for the others.

### Reservoir Computing Performance Evaluation

The prediction errors were evaluated by NMSE for NARMA2 task and NRMSE for Mackey‐Glass and Hénon map tasks. The NMSE and NRMSE were defined by the following equations:

(8) $$\begin{array}{c} NMSE=\frac{\sum_{k=1}^{T}{\left[y_{target}\left(k\right)-y\left(k\right)\right]}^{2}}{T\sigma^{2}\left[y_{target}\left(k\right)\right]}\; \end{array}$$

(9) $$\begin{array}{c} NRMSE=\sqrt{\frac{\sum_{k=1}^{T}{\left[y_{target}\left(k\right)-y\left(k\right)\right]}^{2}}{T\sigma^{2}\left[y_{target}\left(k\right)\right]}}\; \end{array}$$

where *T* and σ are the data length in the test phase and the standard deviation, respectively.

### Nonlinearity Factor

To quantify the nonlinearity of the input‐output relationship of the reservoir, the nonlinearity factor NL which was commonly used to assess the conductance updating linearity of a memristor was utilized. The output voltage of the reservoir *V_out_* (*T*), as a function of the normalized input amplitude *V_in_* was modeled by

(10) $$\begin{array}{c} V_{out}\;\left(T\right)=V\left(1-e^{-\mathrm{NL}\times V_{in}}\right)+V_{out,min}\left(T\right) \end{array}$$

where $V=\frac{V_{out,max}\left(T\right)-V_{out,min}\left(T\right)}{1-e^{-NL}}$. *V*_*out*,*min*_(*T*) is the minimum output voltage, *V*_*out*,*max*_(*T*) is the maximum output voltage and NL is the nonlinearity factor. When NL = 0, the response was perfectly linear. A higher factor indicates a greater degree of nonlinearity.

## Conflict of Interest

The authors declare no conflict of interest.

## Supporting information



Supporting Information

## Data Availability

The data that support the findings of this study are openly available in GitHub at https://github.com/ZeLinM/RC‐circuit‐reservoir, reference number.^[^ ^44^ ^]^
