# Supplementary material for: Versatile and Robust Reservoir Computing with PWM‐Driven Heterogenous R–C Circuits
Source: Adv Sci (Weinh). 2025 May 14;12(29):e16413. doi: 10.1002/advs.202416413 (PMC12362817; doi:10.1002/advs.202416413)
Supplement: Supplementary file 1 — Supporting Information [file ADVS-12-e16413-s001.docx]

Supporting Information

**Versatile and Robust Reservoir Computing with PWM-Driven Heterogenous R-C Circuits**

*Zelin Ma, Huasen Yi, Ziping Zheng, Zhanyi Chen, Weicheng Liu, Yibing Chen, Bojun Cheng*, Chang Cai*, Shusheng Pan* and Jun Ge**

Z. Ma, H. Yi, Z. Zheng, Z. Chen, W. Liu, Y. Chen, Prof. C. Cai, Prof. S. Pan, Prof. J. Ge

School of Physics and Material Science

Guangzhou University

Guangzhou Higher Education Mega Center, Panyu District, Guangzhou, 510006, China

E-mail: [caichang@gzhu.edu.cn](mailto:caichang@gzhu.edu.cn); [sspan@gzhu.edu.cn](mailto:sspan@gzhu.edu.cn); [speegejun510@gzhu.edu.cn](mailto:speegejun510@gzhu.edu.cn)

Z. Ma, Prof. S. Pan, Prof. J. Ge

Research Center for Advanced Information Materials (CAIM)

Huangpu Research & Graduate School of Guangzhou University

Sino-Singapore Guangzhou Knowledge City, Huangpu District, Guangzhou, 510555, China

Prof. J. Ge, Prof. S. Pan

Key Lab of Si-based Information Materials & Devices and Integrated Circuits Design Department of Education of Guangdong Province

Guangzhou Higher Education Mega Center, Panyu District, Guangzhou, 510006, China

Z. Ma, Prof. B. Cheng

Microelectronics Thrust

The Hong Kong University of Science and Technology (Guangzhou)

No. 1 Duxue Road, Nansha District, Guangzhou, 511466, China

E-mail: [bocheng@hkust-gz.edu.cn](mailto:bocheng@hkust-gz.edu.cn)


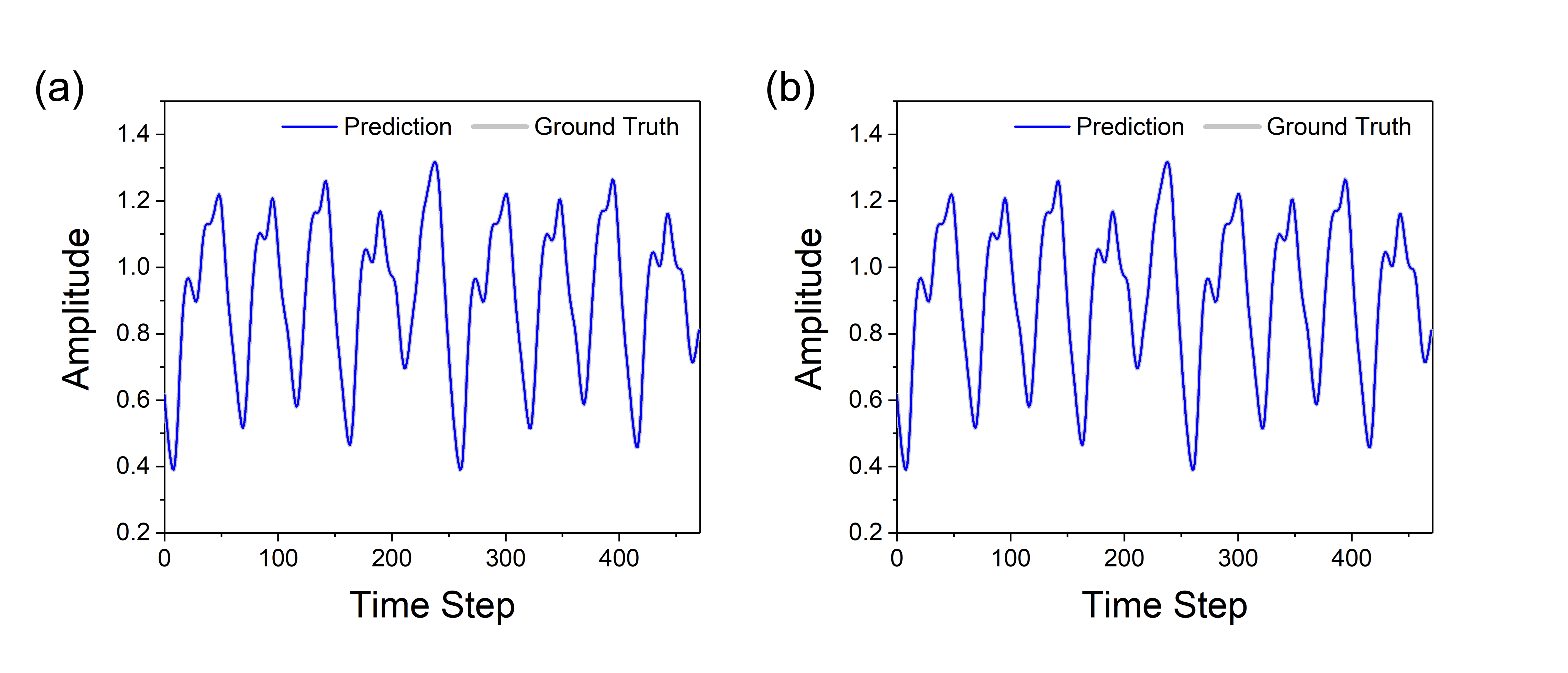


**Figure S1.** **Training process of the R-C circuits reservoir during the first 500 time steps of the Mackey-Glass task.** (a) Training prediction result with an NRMSE of 0.0005 for the grouped reservoir encoded by PAM. (b) Training prediction result with an NRMSE of 0.0008 for the grouped reservoir encoded by PWM. Both encoding methods for the input signals achieved very small one-step prediction errors, with PAM showing even smaller errors compared to PWM.

**Note S1. An illustration of different nonlinearity requirements for different tasks.**

The NARMA2, Mackey-Glass, and Hénon map time series prediction tasks exhibit varying requirements for dynamics, making them ideal benchmarks for RC system performance. To explore this, we designed parallel reservoir systems using R-C circuits, each consisting of 8 identical sub-reservoirs. Each reservoir system has the same time constant ($\tau$) but varying pulse durations ($T$), leading to different levels of nonlinearity. The nonlinearity levels were quantified by the ratio of $T$ to $\tau$, ranging from 0.1 to 10, as shown in Table S1.

As identical R-C reservoirs are used, each sub-reservoir processes inputs with a unique 5-element mask sequence. The combined reservoir states from the 40 (8 × 5) nodes were fed into the readout layer (a 41 × 1 network) to predict the next time step. The prediction errors, as detailed in Table S1, reveal distinct patterns:

For the NARMA2 task, smaller prediction errors were observed at smaller $T/\tau$ ratios, which correspond to lower nonlinearity. This suggests that NARMA2, despite its complexity, can be handled more effectively with reservoirs that exhibit lower nonlinearity. For the Mackey-Glass and Hénon map tasks, better performance was achieved at larger $T/\tau$ ratios, which correspond to higher nonlinearity. This is likely because both tasks involve chaotic systems that require stronger nonlinearity to capture their more complex dynamics.

These findings also demonstrate that a reservoir system with a single nonlinearity configuration cannot adequately address the diverse requirements of tasks with varying degrees of complexity.

**Table S1. Prediction errors of three benchmark tasks for** **different parallel reservoir systems.**

| $\boldsymbol{T}\boldsymbol{/\tau}$ | **NARMA2 Prediction**  **/NMSE** | **Mackey-Glass Prediction /NRMSE** | **Hénon Map Prediction**  **/NRMSE** |
| --- | --- | --- | --- |
| 10.00 | 0.126 | 0.159 | 0.334 |
| 5.00 | 0.111 | 0.049 | 0.118 |
| 2.50 | 0.124 | 0.032 | 0.034 |
| 1.00 | 0.142 | **0.028** | **0.007** |
| 0.63 | 0.135 | 0.029 | 0.011 |
| 0.45 | 0.124 | 0.030 | 0.027 |
| 0.36 | 0.112 | 0.032 | 0.044 |
| 0.30 | 0.101 | 0.034 | 0.061 |
| 0.25 | 0.091 | 0.037 | 0.077 |
| 0.22 | 0.082 | 0.039 | 0.091 |
| 0.13 | 0.054 | 0.049 | 0.143 |
| 0.10 | **0.045** | 0.055 | 0.169 |


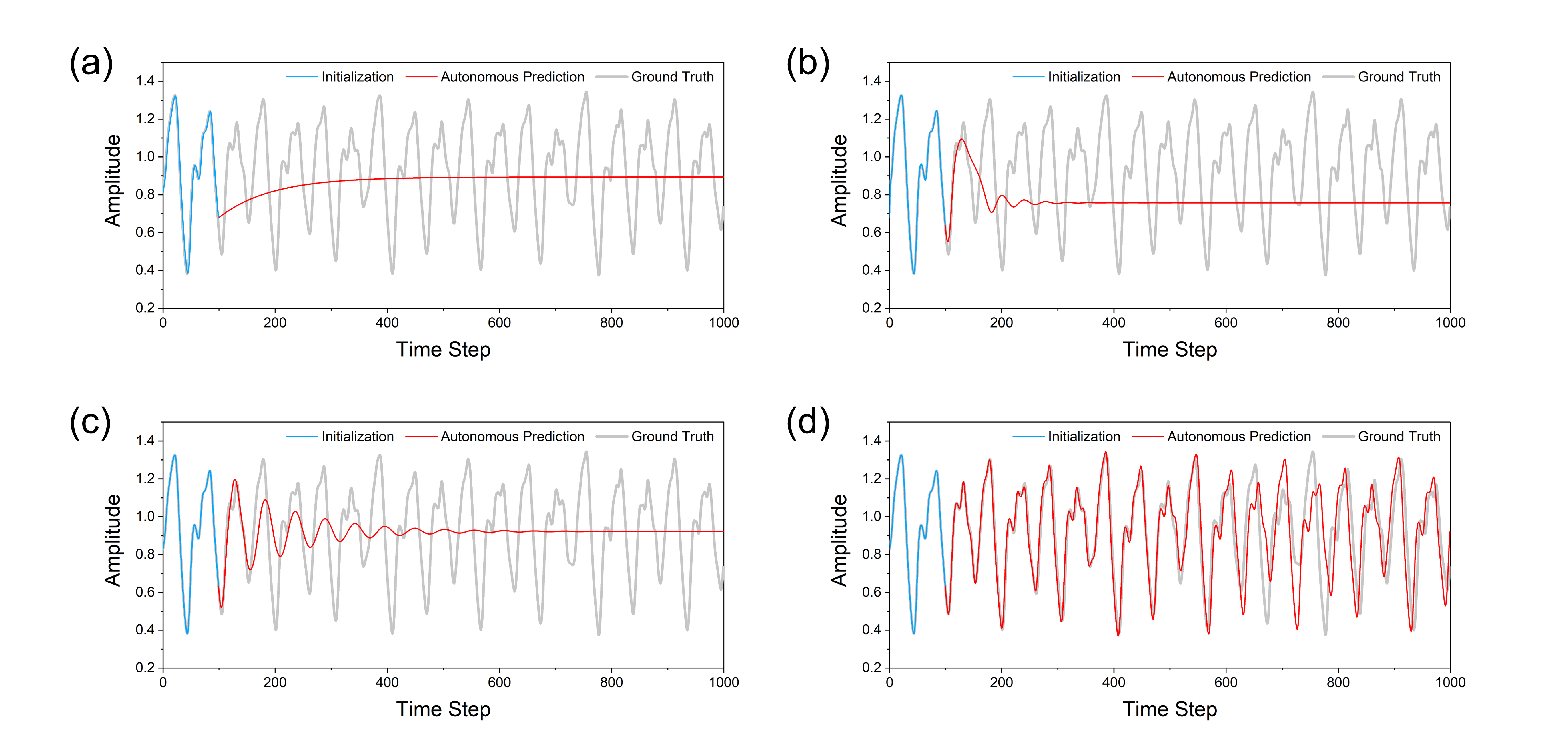


**Figure S2.** **Autonomous prediction results of the grouped reservoir for the Mackey-Glass prediction task after 1000 training time steps.** (a) Autonomous prediction result without reservoir under the virtual node mode (NRMSE = 1.017). (b) Autonomous prediction result of the grouped reservoir under the virtual node mode (NRMSE = 1.032). (c) Autonomous prediction result without reservoir under the intermediate node mode (NRMSE = 0.941). (d) Autonomous prediction result of the grouped reservoir under the intermediate node mode (NRMSE = 0.569). For the virtual node mode, mask length, device number and mask data interval are 30, 8 and 4 ms, respectively. For the intermediate node mode, intermediate node number, device number and data interval are 30, 8 and 4 ms, respectively. Only the grouped reservoir under the intermediate node mode is able to carry out autonomous prediction.


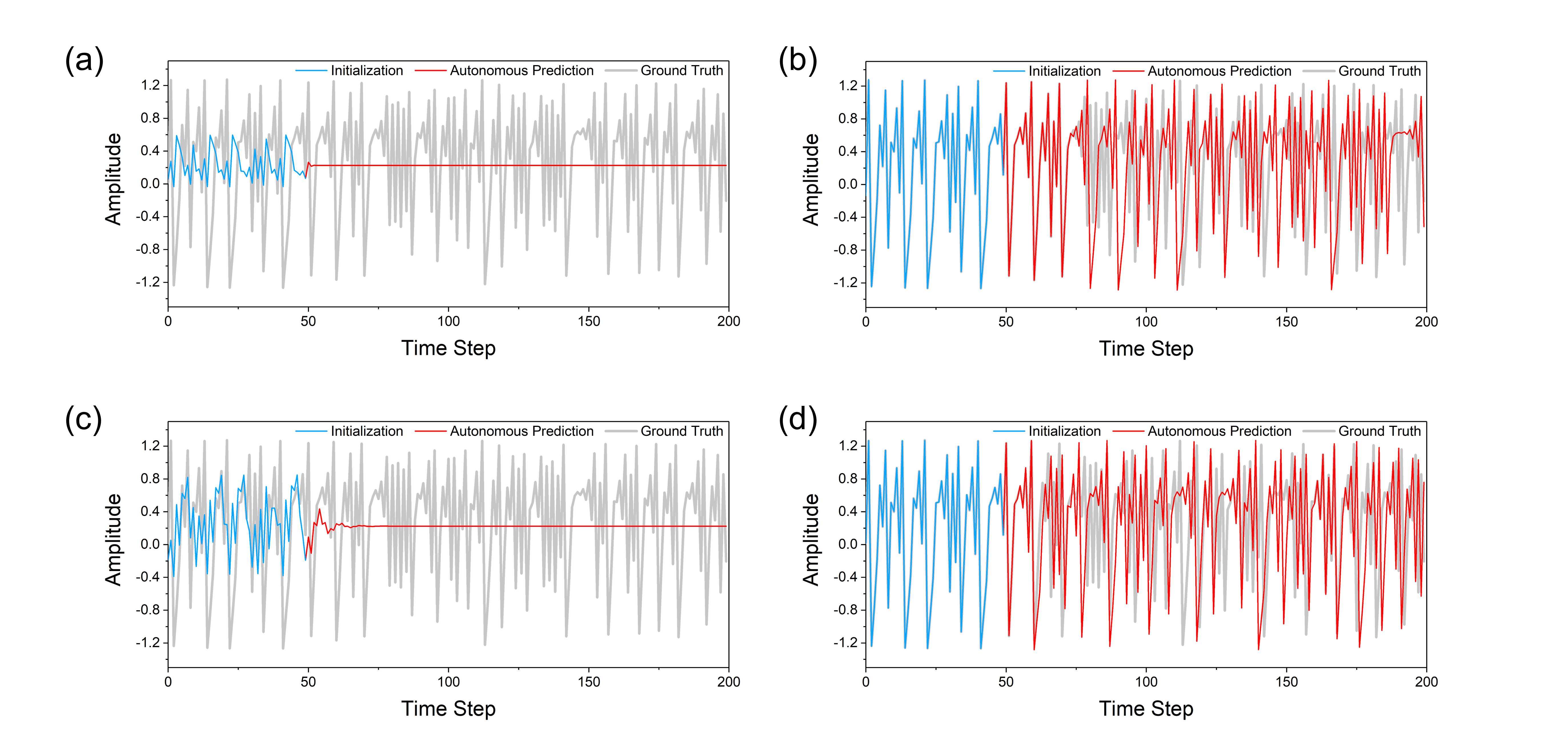


**Figure S3.** **Autonomous prediction results of the grouped reservoir for the Hénon map prediction task after 400 training time steps.** (a) Autonomous prediction result without reservoir under the virtual node mode (NRMSE = 1.004). (b) Autonomous prediction result of the grouped reservoir under the virtual node mode (NRMSE = 1.326). (c) Autonomous prediction result without reservoir under the intermediate node mode (NRMSE = 1.003). (d) Autonomous prediction result of the grouped reservoir under the intermediate node mode (NRMSE = 1.420). For the virtual node mode, mask length, device number and mask data interval are 5, 8 and 4 ms, respectively. For the intermediate node mode, intermediate node number, device number and data interval are 5, 8 and 4 ms, respectively. The grouped reservoirs under both the virtual and intermediate node mode are able to carry out autonomous prediction.

**Table S2. Comparison of reservoir performance of representative state-of-the-art physical reservoirs.**

| **Task** | **Physical node** | **Exp./sim.** | **# of physical nodes** | **# of virtual nodes** | **# of inter-mediate nodes** | **# of total**  **states** | **Error** | **Ref.** |
| --- | --- | --- | --- | --- | --- | --- | --- | --- |
| NARMA2 prediction | Ion-gating reservoir | Exp. | 8 | 10 | / | 80 | NMSE = 0.02 | ^[1]^ |
|  | Nanoparticles network | Exp. | 90 | / | / | 90 | NMSE = 0.008 | ^[2]^ |
|  | Carbon nanotubes network | Exp. | 20 | 2 | / | 40 | NMSE = 0.368 | ^[3]^ |
|  | Carbon nanotubes network | Exp. | 60 | / | / | 60 | NMSE = 0.023 | ^[4]^ |
|  | Electro-chemical Reaction | Exp. | 112 | / | / | 112 | NMSE = 0.0624 | ^[5]^ |
|  | Diode circuits | Exp. | 300 | / | / | 300 | NMSE = 0.077 | ^[6]^ |
|  | Hf_0.5_Zr_0.5_O_2_ transistors | Exp. | 25 | 50 | / | 1250 | NMSE = 0.0092 | ^[7]^ |
|  | This work | Exp. | 8 | / | 5 | 40 | NMSE = 0.002 | / |
|  | This work | Sim. | 8 | / | 5 | 40 | NMSE = 0.001 | / |
| Mackey-Glass prediction | Rotating neurons | Exp. | 64 | / | / | 64 | NRMSE = 0.03 | ^[8]^ |
|  | Strain-mediated  Spintronic devices | Exp. | 50 | / | 31 | 1550 | NRMSE = 0.16 | ^[9]^ |
|  | Hf_0.5_Zr_0.5_O_2_ transistors | Exp. | 25 | 50 | / | 1250 | NRMSE = 0.008 | ^[7]^ |
|  | CuInP_2_S_6_ memristors | Exp. | 4 | 10 | / | 40 | NRMSE = 0.014 | ^[10]^ |
|  | This work | Exp. | 8 | / | 5 | 40 | NRMSE = 0.015 | / |
|  | This work | Sim. | 8 | / | 5 | 40 | NRMSE = 0.004 | / |
| Hénon map prediction | NbO_x_ memristors | Sim. | 100 | / | / | 100 | NRMSE = 0.07 | ^[11]^ |
|  | TiO_x_/TaO_y_ memristors | Exp. | 25 | 4 | / | 100 | NRMSE = 0.046 | ^[12]^ |
|  | Ferroelectric diodes | Exp. | 8 | 3 | 24 | 48 | NRMSE = 0.017 | ^[13]^ |
|  | CuInP_2_S_6_ memristors | Exp. | 4 | 10 | / | 40 | NRMSE = 0.048 | ^[10]^ |
|  | Hf_0.5_Zr_0.5_O_2_ transistors | Exp. | 25 | 50 | / | 1250 | NRMSE = 0.000585 | ^[7]^ |
|  | This work | Exp. | 8 | / | 5 | 40 | NRMSE = 0.021 | / |
|  | This work | Sim. | 8 | / | 5 | 40 | NRMSE = 0.004 | / |

**Note S2. Dynamic memristor model.**

The dynamic memristor model in this article is inspired by references^[14-15]^. The conductance of the memristor and its evolution over time was regulated by means of a physics-based potentiation-depression rate balance equation. The current $I$ flowing in device under the applied voltage $V$ is described by the relation:

$$\begin{aligned} I=\left[ G_{\min}\left( 1-g \right)+G_{\max}g \right]V\#\left( 1 \right) \end{aligned}$$

where 0 ≤ $g$ ≤ 1 is the normalized conductance. $G_{\max}$ and $G_{\min}$ are the maximum and minimum conductances, respectively, used for model calibration. We set $G_{\max}$ and $G_{\min}$ to 10^-3^ S and 10^-9^ S. The state equation describing short-term plasticity of the device can be expressed by the balance equation:

$$\begin{aligned} \frac{dg}{dt}=\kappa_{P}\left( V \right)\left( 1-g \right)-\kappa_{D}\left( V \right)g\#\left( 2 \right) \end{aligned}$$

where $\kappa_{P}\left( V \right)$ and $\kappa_{D}\left( V \right)$ are the potentiation and depression rate coefficients that are assumed to be function of the applied voltage through exponential relations, as expected for diffusion of ions:

$$\begin{aligned} \kappa_{P}\left( V \right)=\kappa_{P0}\exp\left( +V\eta_{P} \right)\#\left( 3 \right) \end{aligned}$$

$$\begin{aligned} \kappa_{D}\left( V \right)=\kappa_{D0}\exp\left( -V\eta_{D} \right)\#\left( 4 \right) \end{aligned}$$

where $\kappa_{P0}$, $\kappa_{D0}$ > 0 are constants and are set to 10^-5^ s^-1^ and 2000 s^-1^, respectively. $\eta_{P}$, $\eta_{D}$ > 0 are transition rates and are set to 26 and 15, respectively. Equation (2) can be recursively solved as (assuming a simulation timestep $\Delta t$ > 0):

$$\begin{aligned} g_{t}=\frac{\kappa_{P}}{\kappa_{P}+\kappa_{D}}\left\{ 1-\left[ 1-\left( 1+\frac{\kappa_{D}}{\kappa_{P}} \right)g_{t-1} \right]e^{-\left( \kappa_{P}+\kappa_{D} \right)\Delta t} \right\}\#\left( 5 \right) \end{aligned}$$

where $g_{t}$ and $g_{t-1}$ are normalized conductances at times $t$ and $t-1$ of the device. Note that Equation (5) is not expressed as a continuous function of $t$ and allows to simulate the response of the dynamic memristor for an arbitrary input voltage (including voltage pulses).

Figure S4a-c show the analog I-V characteristics, fading memory and nonlinearity properties of the simulated dynamic memristor, which are similar to those reported in the literatures^[12]^. The characteristic decay time constant of the dynamic memristor is about 1 ms. The SET voltage (V_set_) of the device can be contronlled by $\eta_{P}$ (V_set_ ≈ -ln($\kappa_{P0}$)/ $\eta_{P}$).

Similar to the grouped R-C circuits reservoirs, the memristor-based reservoir consists of eight parallel devices. The device-to-device variation in the V_set_ of the memristors is simulated by adding Gaussian noise to the V_set_ of each device. The variation level is quantified by the coefficient of variation (C_v_), which is defined as the standard deviation of the added Gaussian noise divided by the mean V_set_. In this study, the C_v_ is set to 5%, a commonly observed value in the literature^[16-18]^. To assess copy-to-copy variation, memristors reservoir A and memristors reservoir B are designed with the same device-to-device C_v_, but with different values of V_set_. The readout layer weights are trained using data from memristor reservoir A and are kept unchanged during the testing phase with memristors reservoir B. The intermediate node mode and the number of nodes are consistent with those used in the R-C circuits reservoirs. All input pulse sequences applied to the dynamic memristor model have pulse amplitudes ranging from 0.3 to 0.5 V (amplitude encoding), with a pulse width of 500 µs and a pulse interval of 200 µs.


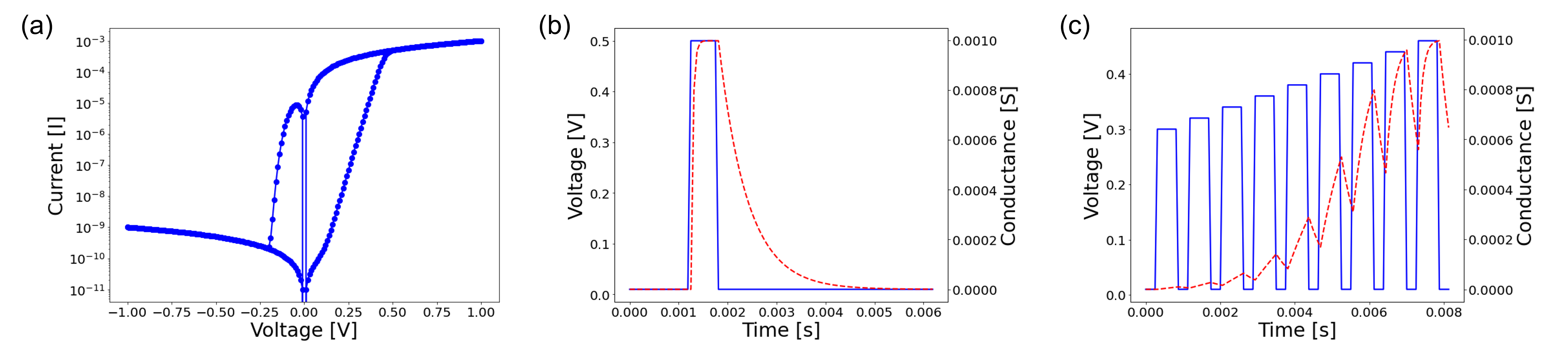


**Figure S4.** **Electrical characteristics of the simulated dynamic memristor.** (a) I-V curve of deive. (b) The Evolution of the device conductance with time during the application of a write pulse (0.5 V, 500 µs), showing short-term memory characteristics. Read voltage of 0.01 V is applied before and after the write pulse. (c) The Evolution of the device conductance with time during the application of a train of write pulses with pulse amplitudes from 0.3 V to 0.45 V, pulse width of 500 µs and pulse interval of 200 µs, showing nonlinear conductance changes. Read voltage of 0.01 V is applied between the write pulses.

**Note S3. Integration-ReLU-leakage circuit model.**

The circuit simulation was carried out using Cadence Virtuoso. The Integration-ReLU-leakage circuit shown in Figure S5a, is an analog circuit that has been used in physical reservoir systems^[8]^. This circuit meets the requirements for nonlinearity and fading memory, which are essential for a neuron in a reservoir. In this circuit, C_int_ and R_int_ (10 kΩ) act as integrators, while the rectifying diode D_ReLU_ provides a nonlinear activation function, similar to a ReLU function. The diode D_ReLU_ is a germanium diode with a forward voltage of approximately 0.3 V. A relatively high resistance R_leakage_ (100 kΩ) is incorporated to control the current leakage rate.

Similar to the grouped R-C circuits reservoirs, this reservoir consists of eight parallel integration-ReLU-leakage circuits. To introduce device-to-device variation, different capacitance values for C_int_, ranging from 0.6 µF to 2.0 µF, were applied across the circuits. It should be noted that changing the capacitance only affects the fading memory, while the nonlinearity remains unchanged. This is because the nonlinearity is controlled by the rectifying diode, which cannot be continuously adjusted.

The input-output relationship of the circuit is presented in Figure S5b, illustrating the nonlinearity. Due to the high temperature sensitivity of the diode, the input-output characteristics of the reservoir at 80 °C are noticeably different from those at 25 °C. This temperature sensitivity is likely to cause significant performance degradation in the reservoir system.

To isolate the effect of temperature on the reservoir’s performance, the readout layer weights were trained using data from the ReLU circuit reservoir A at RT and remained fixed during the testing phase at 80 °C. The intermediate node configuration and the number of nodes were consistent with those used in the R-C circuits reservoirs. All input pulse sequences applied to the circuit had amplitudes ranging from -1 V to 3 V (amplitude encoding), with a pulse width of 3 ms and no pulse interval.


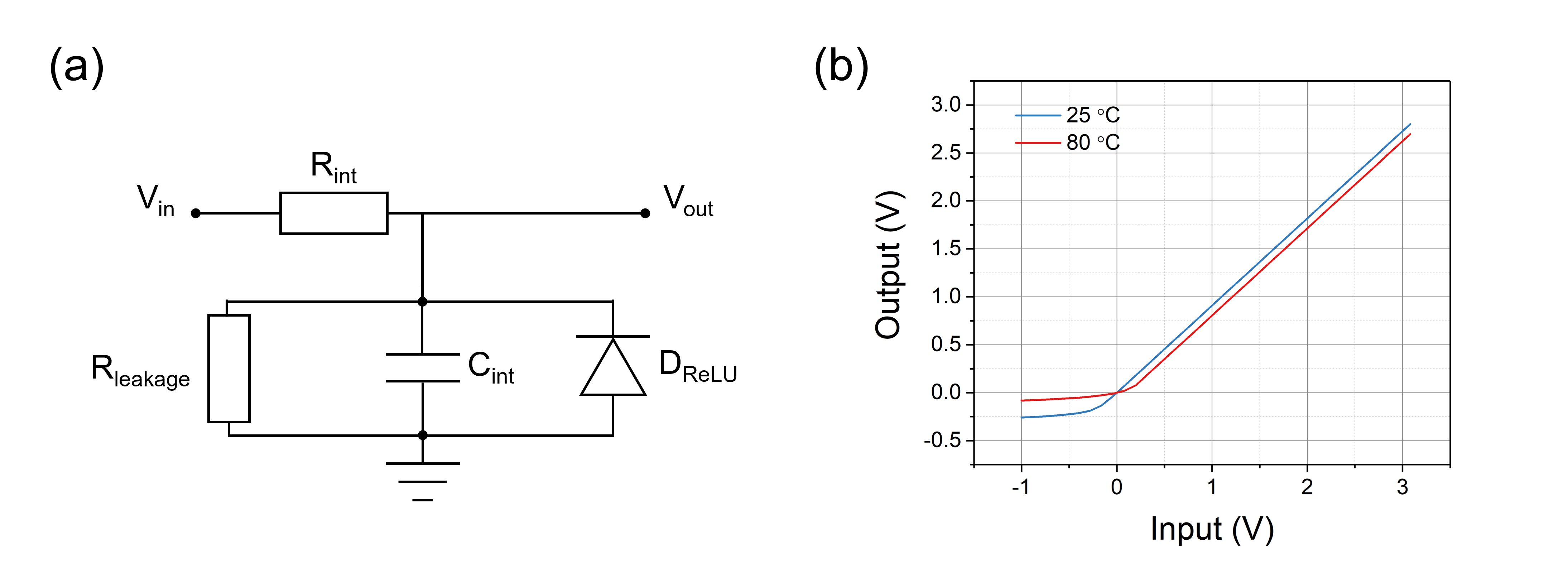


**Figure S5.** **Structure and nonlinear property of the integration-ReLU-leakage circuit.** (a) Schematic diagram of the circuit**.** (b) Input-output relationship of the circuit at 25 ℃ and 80 ℃.


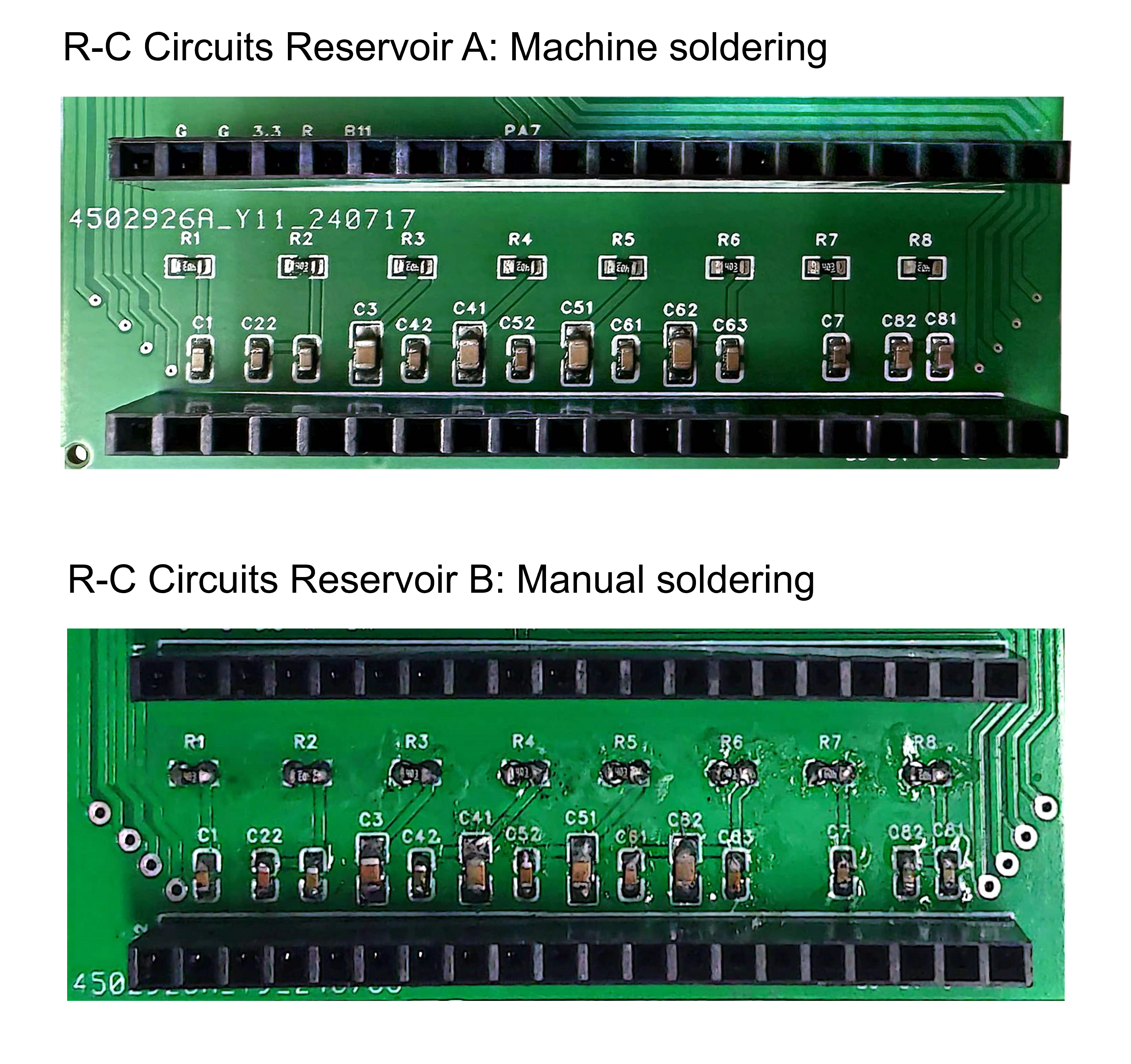


**Figure S6.** **Photograph of two R-C circuits reservoirs labeled as A and B.** Circuit A was assembled using machine soldering, while circuit B was manually assembled using hand soldering techniques.


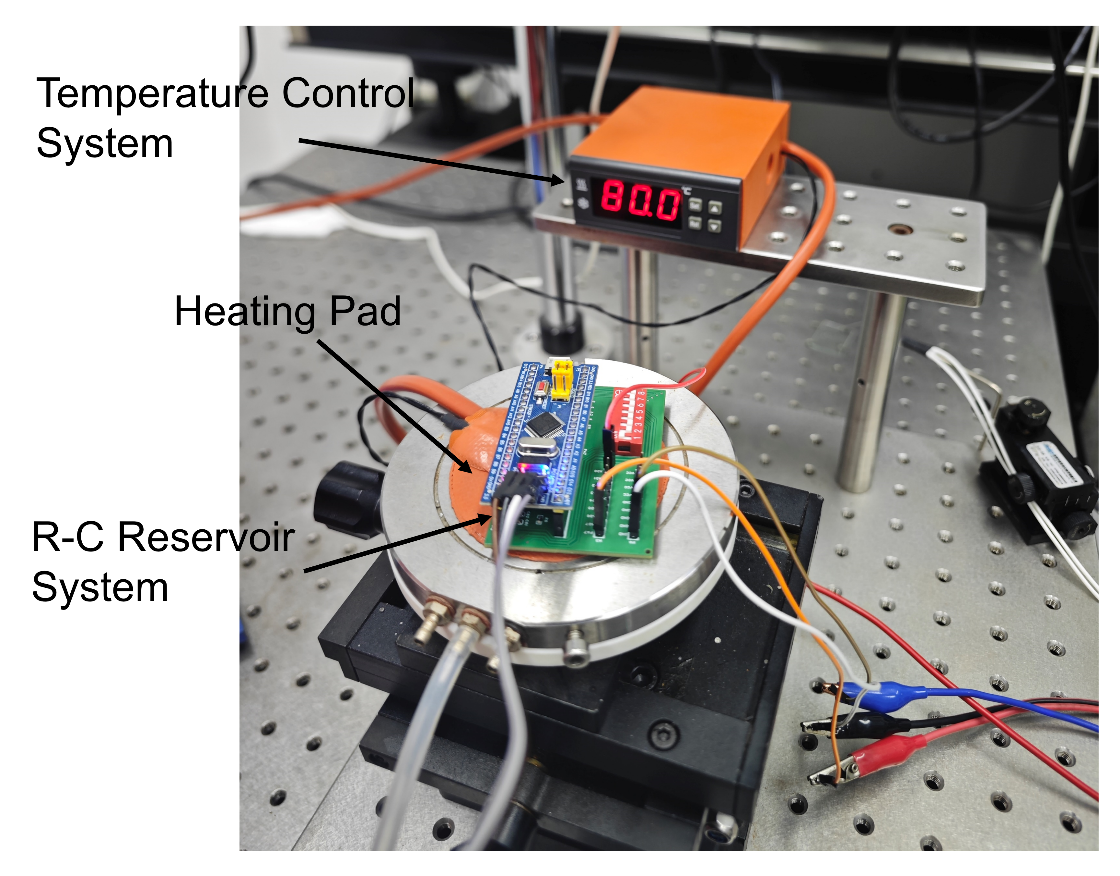


**Figure S7.** **Photograph of the setup for testing the R-C circuits reservoir under 80 °C conditions.**


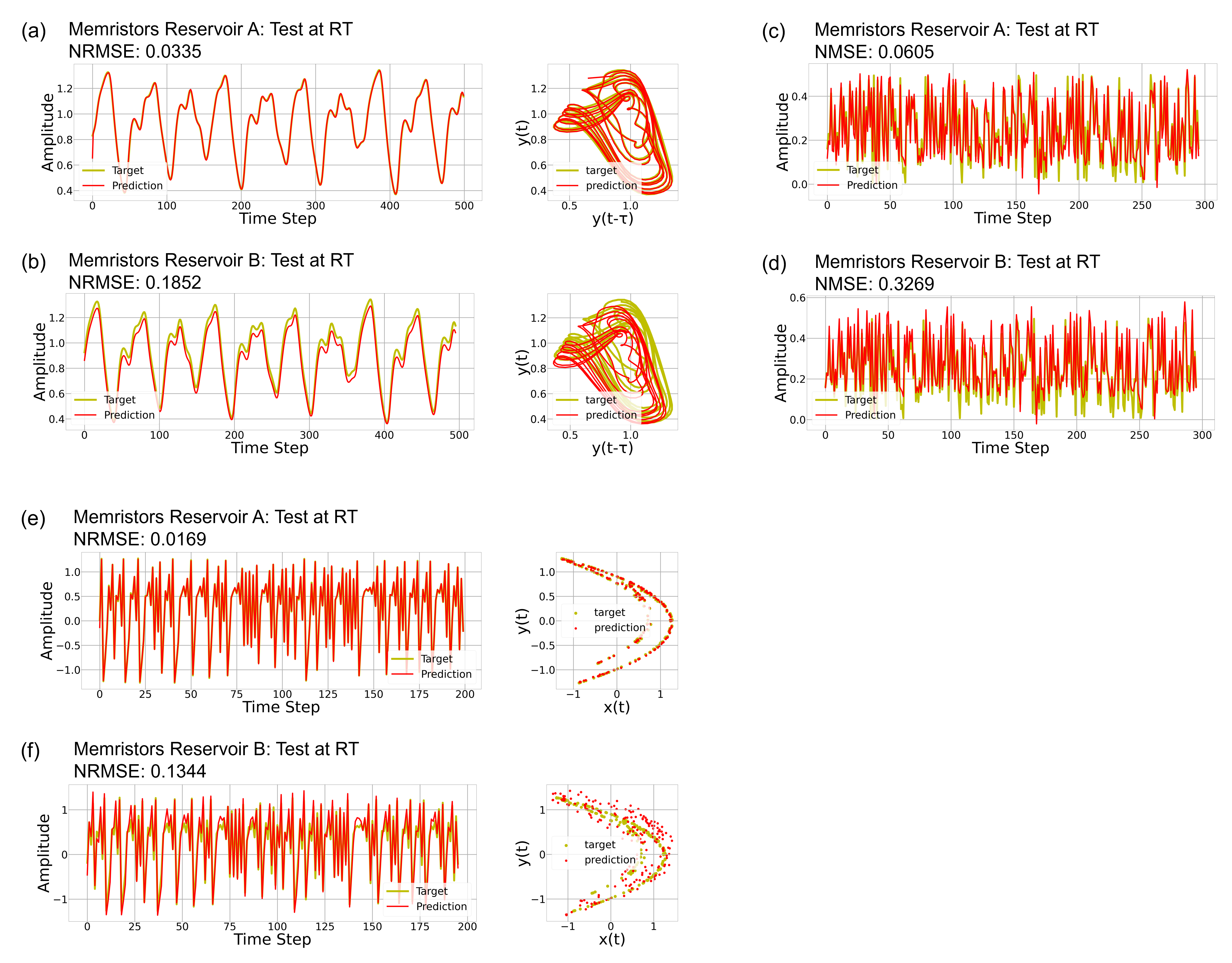


**Figure S8.** **Test prediction results of the memristors reservoir A and B at RT for the Mackey-Glass (a-b), NARMA2 (c-d) and Hénon map (e-f) time series, respectively.**


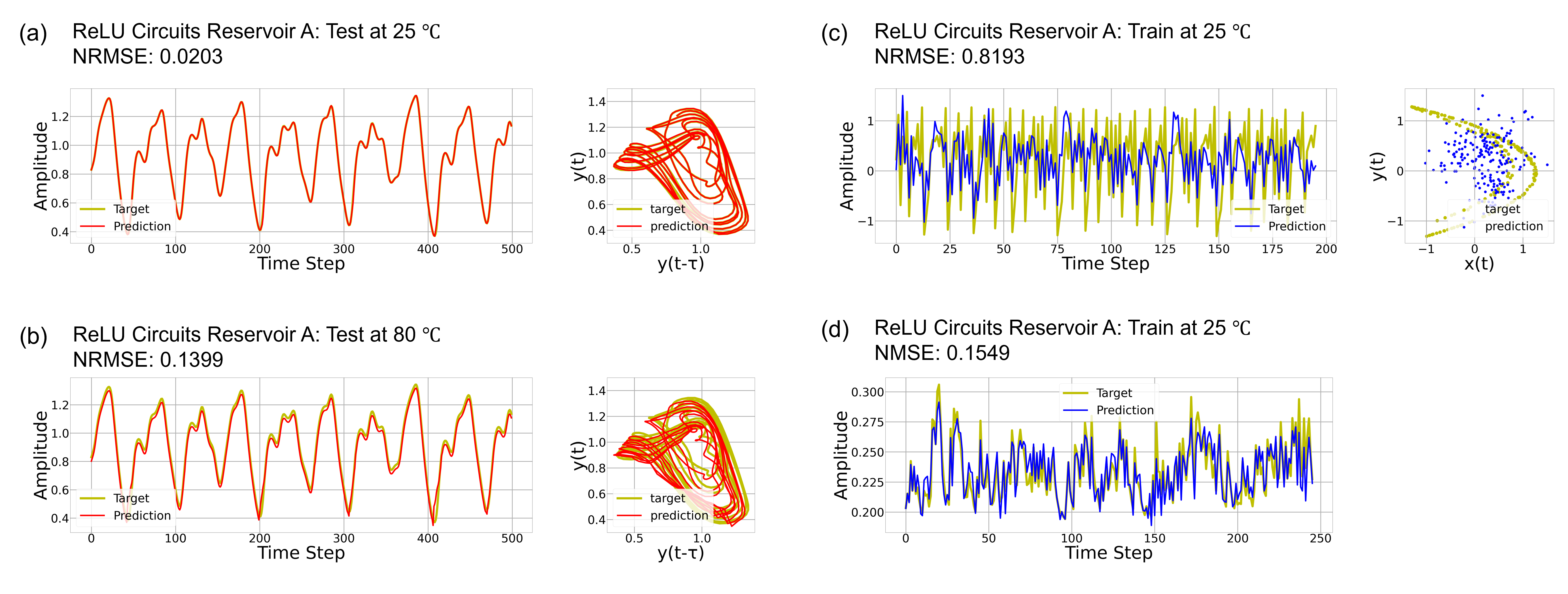


**Figure S9.** (a-b) Test prediction results of the ReLU circuits reservoir A at RT and 80 ℃ for the Mackey-Glass time series. (c-d) Train prediction results of the ReLU circuits reservoir A at RT for the Hénon map and NARMA2 time series. It is important to note that the training processes for the Hénon map and NARMA2 tasks exhibit high prediction errors. Consequently, testing at 80 ℃ was not conducted in Figure 6 of the main text. As discussed in Note S1, these three tasks require different levels of nonlinearity of the reservoir system. However, in the case of the ReLU circuit-based reservoir, adjusting the capacitance only influences the fading memory while leaving the nonlinearity unchanged. This is because the nonlinearity is governed by the rectifying diode, which is difficult to continuously adjust. This limitation may account for the high prediction errors observed in the Hénon map and NARMA2 tasks.


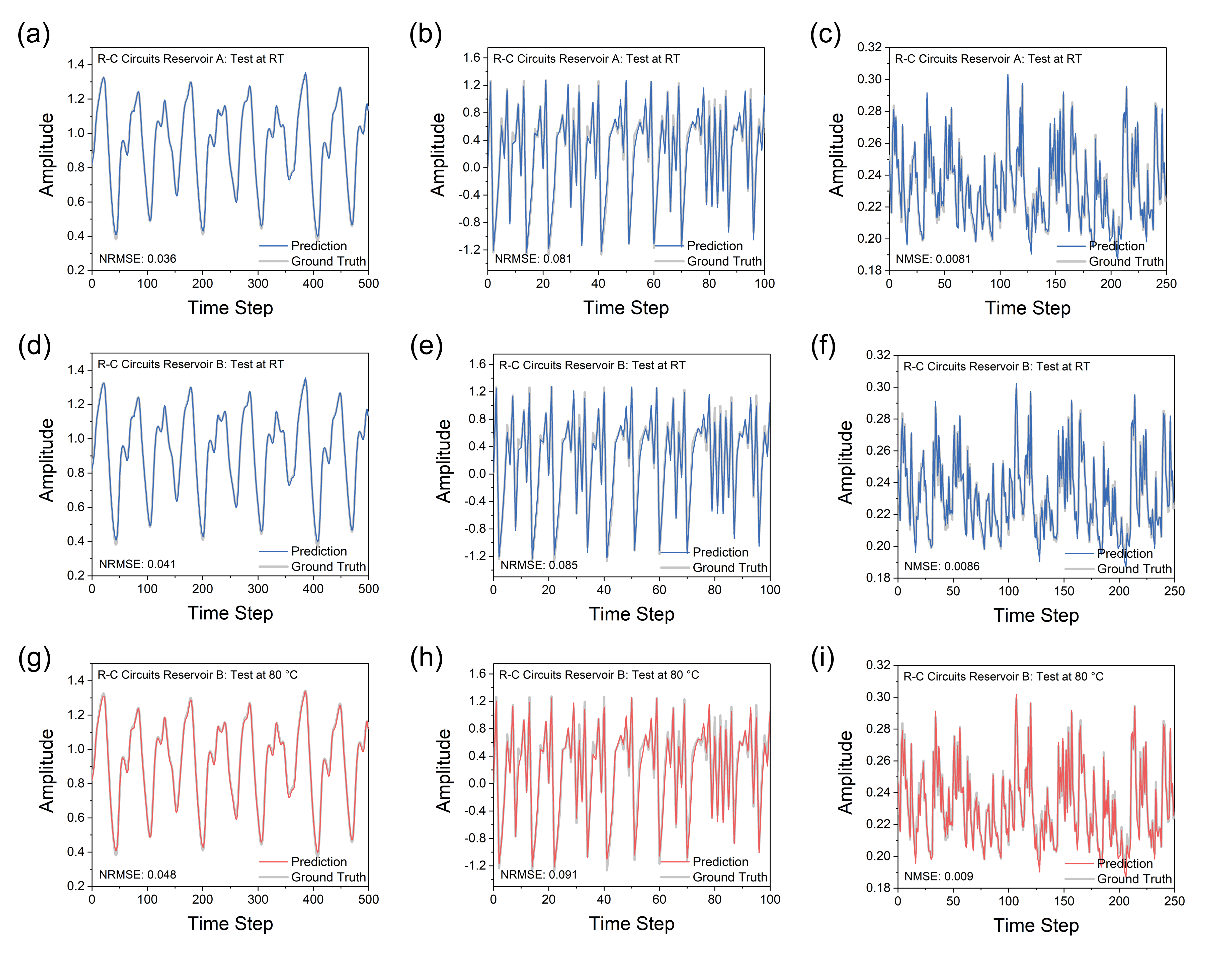


**Figure S10.** **Test** **prediction results of the R-C circuits reservoir A and B at RT and 80 ℃ for the Mackey-Glass, Hénon map and NARMA2 time series, respectively.**

**Table S3. General comparison of physical reservoirs with different technologies.**

| Building Blocks | Customization | Copy-to-copy Stability | Environmental Stability | Footprint | Energy Consumption | Technological Maturity |
| --- | --- | --- | --- | --- | --- | --- |
| Memristor  ^[19-21]^ | Low | Low | Low | **Low** | **Low** | Medium |
| Memristive  Field-effect Transistor  ^[22-25]^ | Medium | Low | Medium | Medium | **Low** | Low |
| Nonlinear  Analog Circuits  ^[8, 26]^ | Medium | **High** | Low | High | High | **High** |
| R-C Circuits  (This Work) | **High** | **High** | **High** | Medium | **Low** | **High** |

**Note S4. Multimode in-sensor RC system based on R-C circuits.**

Multimode in-sensor reservoir computing (MiSRC) advances toward human-like intelligence by mimicking how the brain processes diverse sensory inputs simultaneously. Unlike traditional reservoir computing (RC), which handles single-mode data, MiSRC processes multiple sensor modalities in parallel, closely resembling the brain’s ability to integrate and respond to different types of information (visual, auditory, tactile, olfactory, and painful) in real-time. This multimodal capability, combined with reduced latency and power consumption, allows MiSRC to efficiently process complex, dynamic environments—an essential trait for developing more adaptive and human-like intelligent systems, particularly in biomimetic robot applications (Figure S11a).

Figure S11b illustrates an example of MiSRC based on modified R-C circuits. In this setup, the resistor $R$ is replaced by a series of parallel resistors, each functioning as a specific sensory component. For instance, photoresistors can be used for vision, gas sensitive resistors for smell, and thermistors for heat-induced pain. External stimuli alter the resistance $R_{i}$ ($i$ = 1, 2, 3, ...) of the corresponding device, which in turn modifies the voltage response of the R-C circuit.

$\frac{1}{R_{total}}=\frac{1}{R_{1}}+\frac{1}{R_{2}}+\frac{1}{R_{3}}+\ldots(6)$

$\begin{aligned} \tau_{x}=R_{total}*C\#\left( 7 \right) \end{aligned}$

$$\begin{aligned} V_{out}\left( T \right)=V_{const}\left( 1-e^{-\frac{t}{\tau_{x}}} \right)e^{-\frac{T-t}{\tau_{x}}}\#\left( 8 \right) \end{aligned}$$

Here, $t$ corresponds to the PWM signal from acoustic or pressure sensors. The input $t$ is encoded within the range of $t_{min}$ to $T$​, ensuring that $t_{min}$ is not zero so that the MiSRC can still operate when no stimuli are received from these sensors. This multimodal approach allows the system to process diverse types of signals in a unified and highly fused manner.


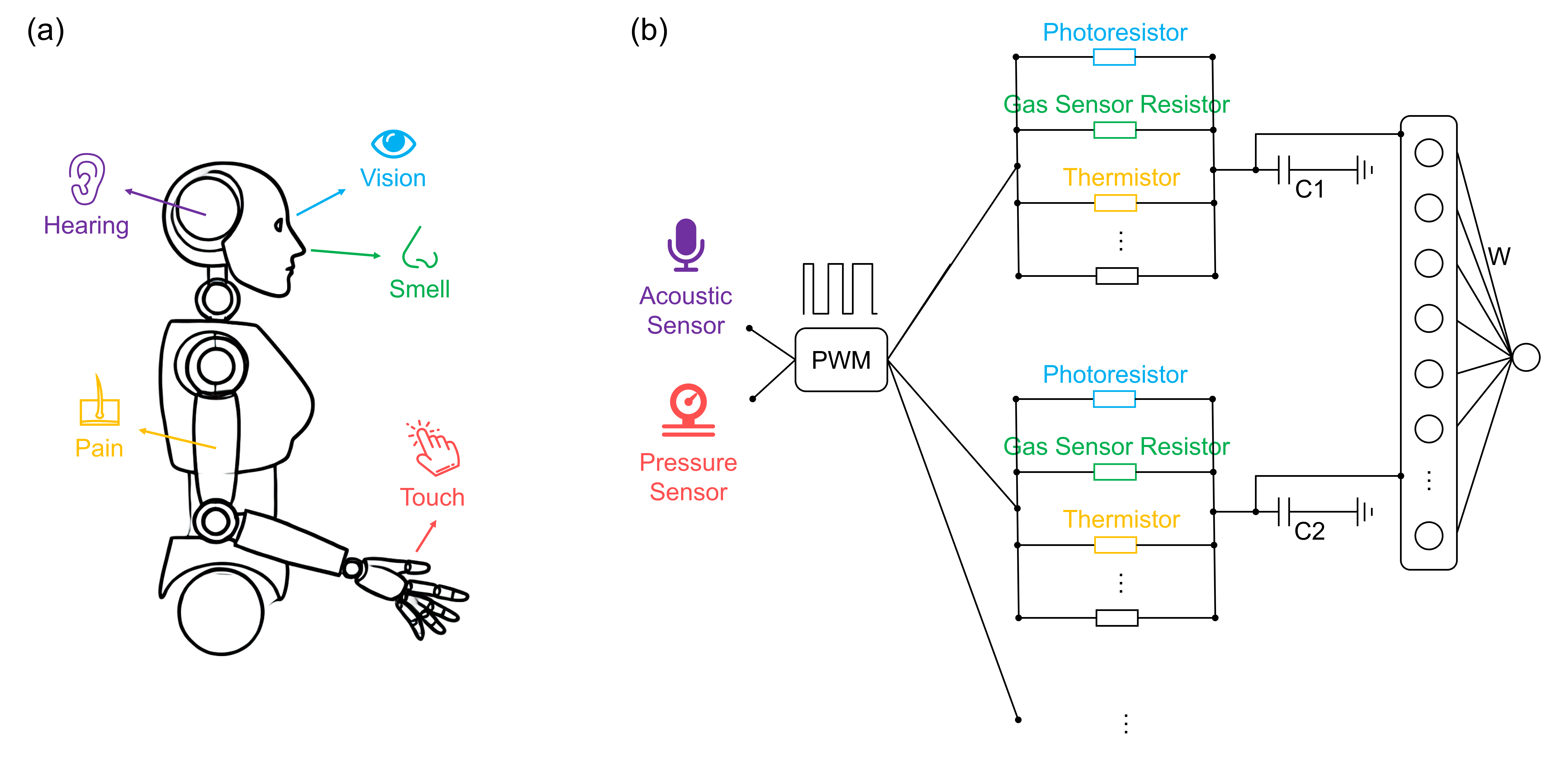


**Figure S11.**  **Schematic of Multimode RC system based on R-C circuits.** (a) Types of sensory input signals in biomimetic robots. (b) Diagram of the multimode R-C circuit reservoir system integrating various sensory signals.

**Note S5. Explanation of the usage of virtual and intermediate nodes in this work.**

Table S4 shows the summary of node selection strategies in this work. While the main text utilizes intermediate nodes for prediction tasks and virtual nodes for classification tasks, it should be noted that both approaches are commonly used for generating reservoir states and are interchangeable for either task type. The key distinction lies in their memory handling: intermediate nodes inherently incorporate historical data responses, thereby enhancing the reservoir's memory capacity at the expense of requiring external memory resources.

On the other hand, to demonstrate how different prediction tasks require distinct nonlinearities (Note S1), we implemented wide reservoir systems comprising eight identical sub-reservoirs. The virtual node method proves particularly effective here, as applying different mask sequences generates diverse outputs - enabling optimal accuracy when paired with appropriate nonlinearities. Conversely, the intermediate node method's lack of mask operations means identical sub-reservoirs produce identical outputs, significantly reducing effectiveness even with the task-appropriate nonlinearity.

**Table S4. Overview of the application of virtual and intermediate nodes in different tasks presented in this work.**

| **Tasks** | **Virtual nodes** | **Intermediate nodes** | **R-C circuit configuration** | **Mask configuration** |
| --- | --- | --- | --- | --- |
| NARMA2 prediction | / | √ | 8 R-C circuits with varied time constants | / |
| Mackey-Glass prediction | / | √ | 8 R-C circuits with varied time constants | / |
| Hénon map prediction | / | √ | 8 R-C circuits with varied time constants | / |
| Multiclass arrhythmic heartbeat classification | √ | / | 8 R-C circuits with varied time constants | 8 identical mask sequences |
| Nonlinearity requirement illustration (Note S1) | √ | / | 8 R-C circuits with identical time constants | 8 varied mask sequences |


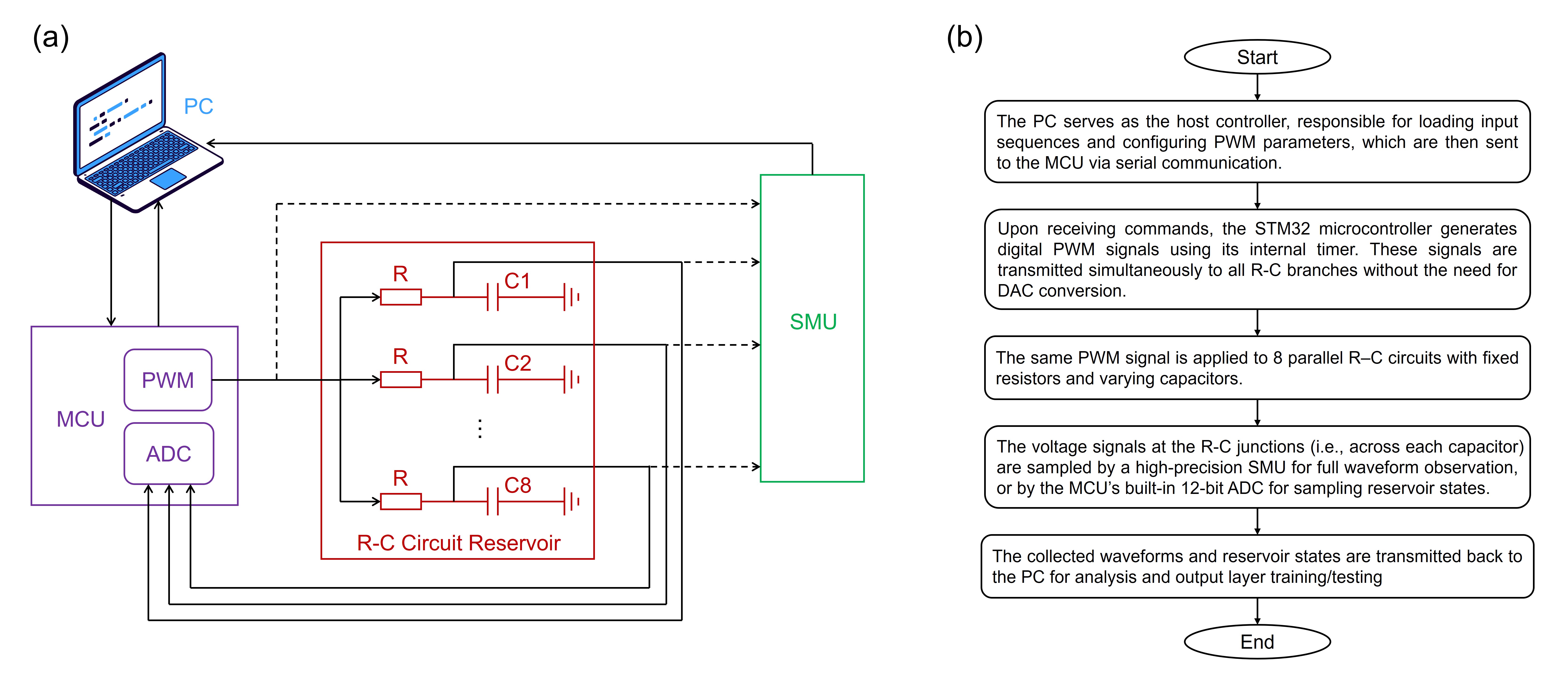


**Figure S12. Circuit schematic (a) and operational workflow (b) of the R-C circuit-based reservoir computing system.**


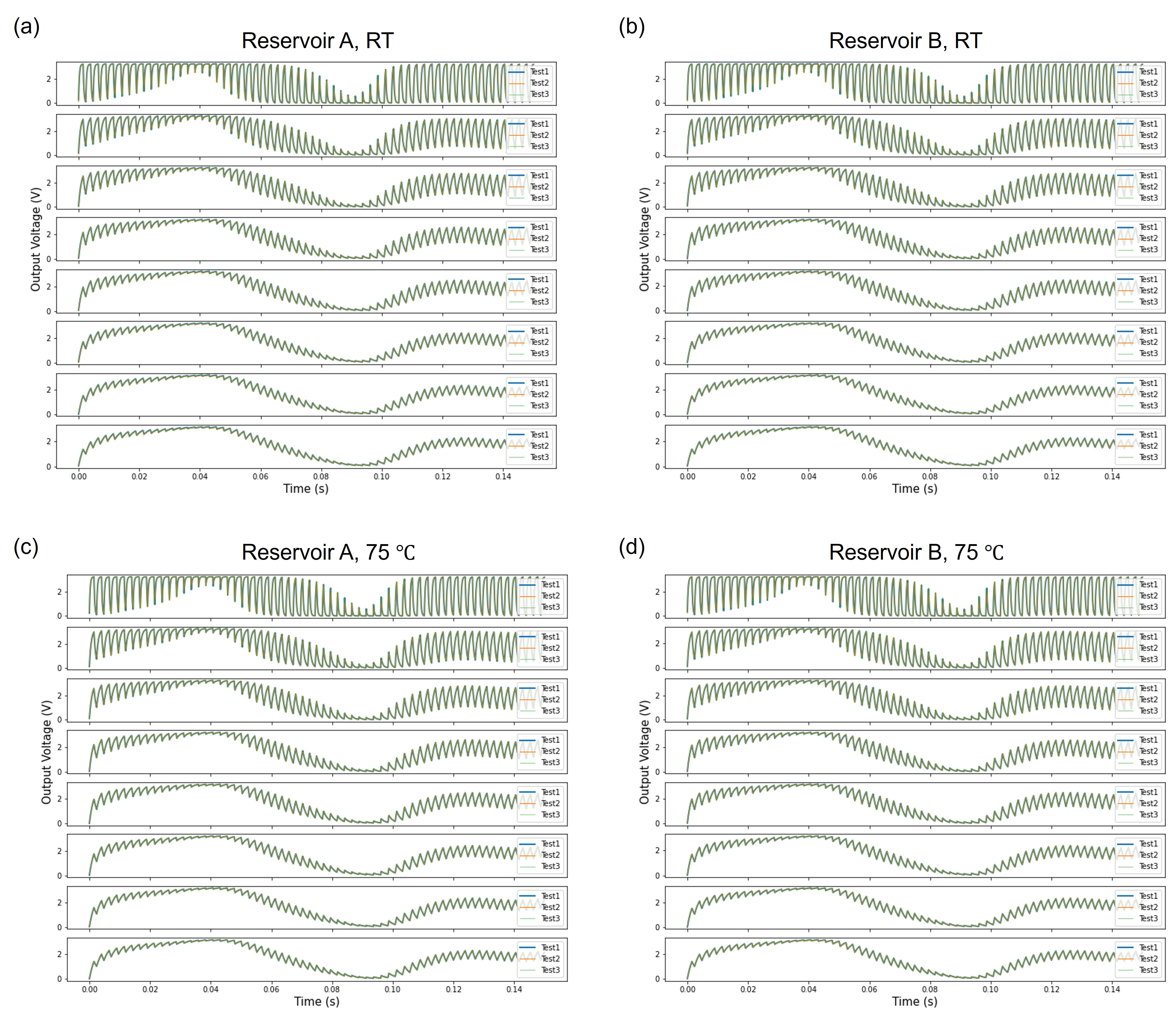


**Figure S13. A subset of raw voltage outputs from the 8-channel R-C circuits for Mackey-Glass prediction task.** Each channel was tested in three repeated trials, with the resulting traces plotted in different colors over 125 PWM periods. The task was conducted on two different reservoirs: Reservoir A, assembled via machine soldering, and Reservoir B, assembled via hand soldering. Both reservoirs were tested under room temperature and elevated temperature (75 °C) conditions.


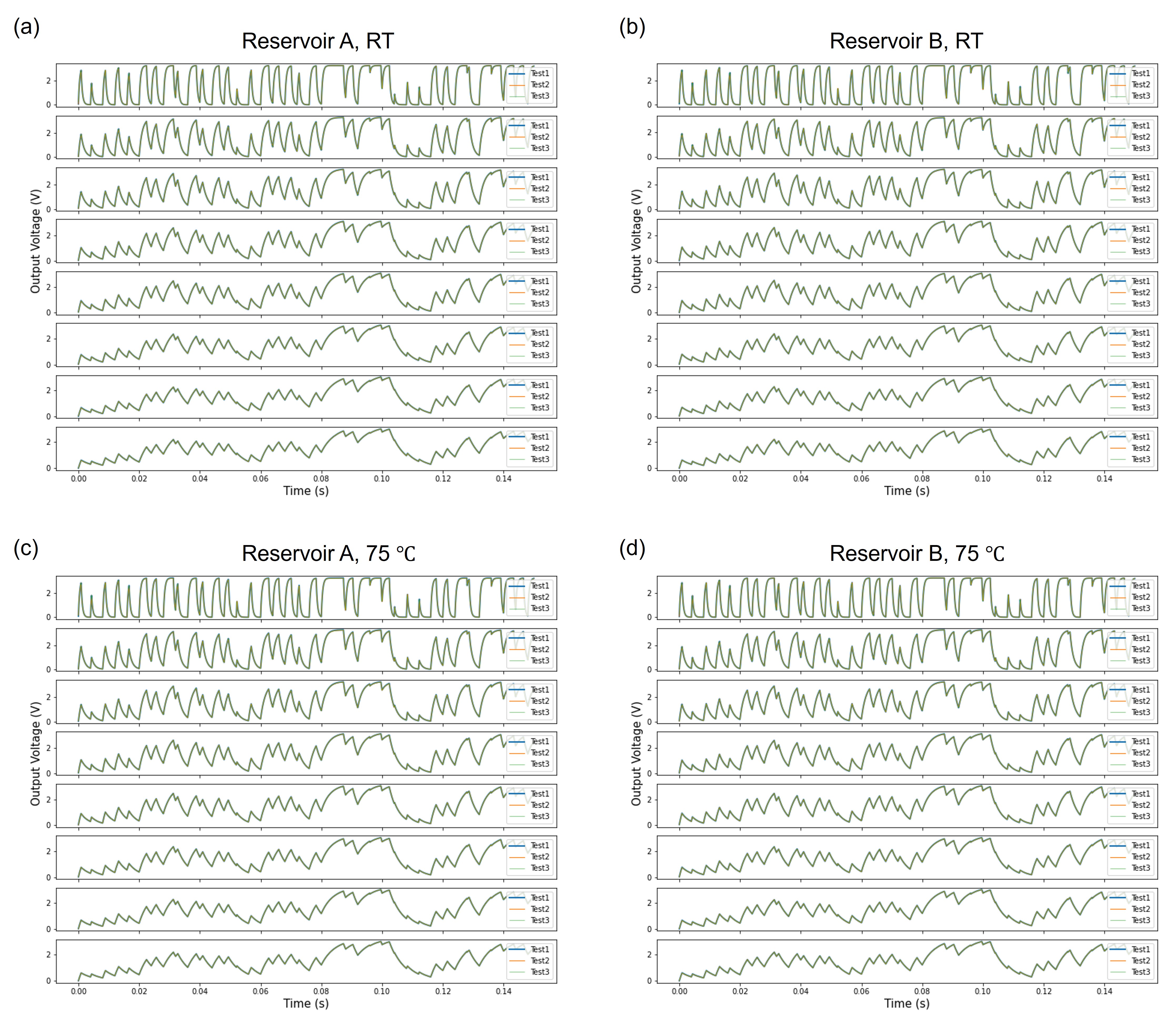


**Figure S14. A subset of raw voltage outputs from the 8-channel R-C circuits for NARMA2 prediction task.** Each channel was tested in three repeated trials, with the resulting traces plotted in different colors over ~37 PWM periods. The task was conducted on two different reservoirs: Reservoir A, assembled via machine soldering, and Reservoir B, assembled via hand soldering. Both reservoirs were tested under room temperature and elevated temperature (75 °C) conditions.


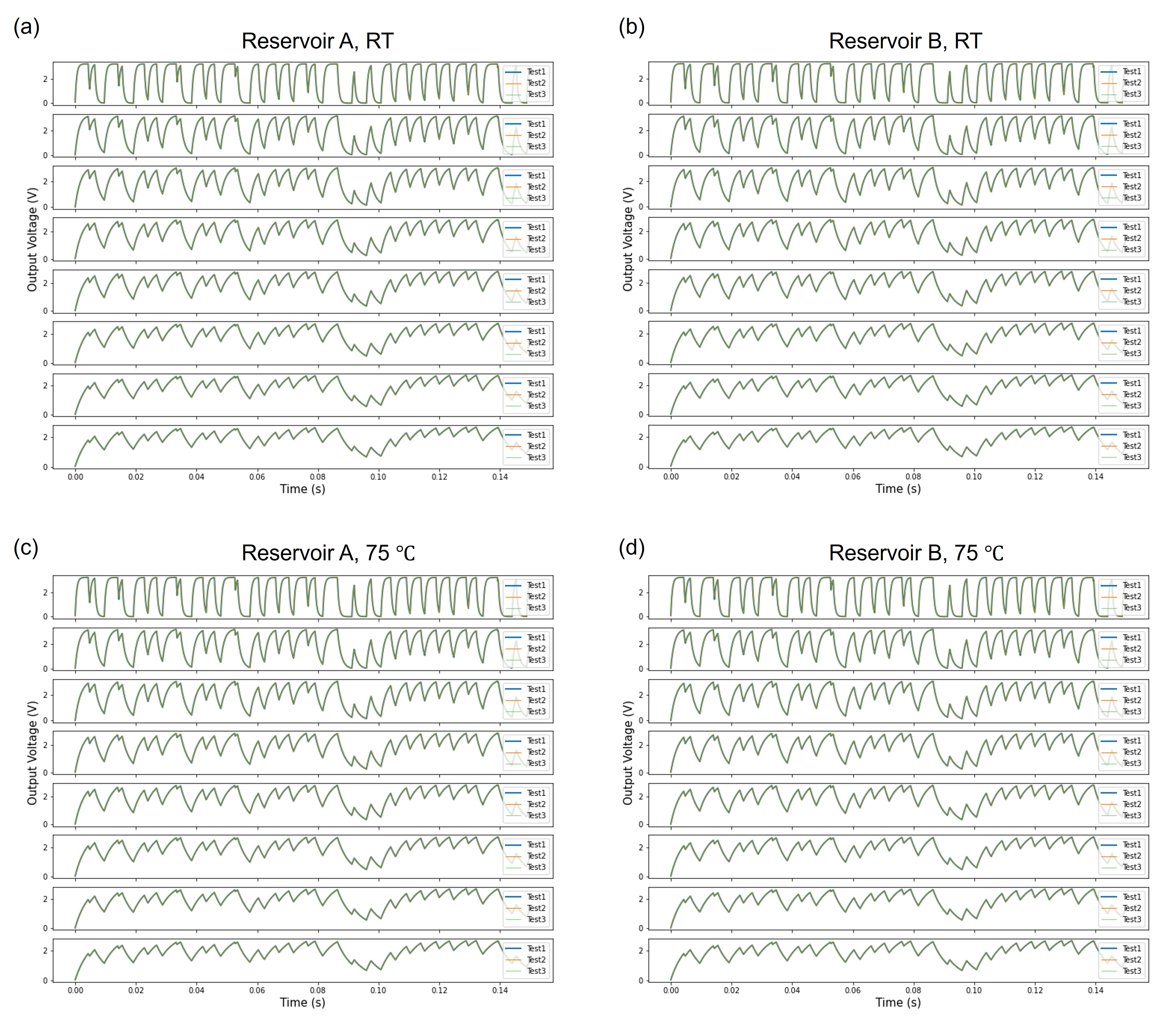


**Figure S15. A subset of raw voltage outputs from the 8-channel R-C circuits for Hénon map prediction task.** Each channel was tested in three repeated trials, with the resulting traces plotted in different colors over 50 PWM periods. The task was conducted on two different reservoirs: Reservoir A, assembled via machine soldering, and Reservoir B, assembled via hand soldering. Both reservoirs were tested under room temperature and elevated temperature (75 °C) conditions.

**References**

[1] D. Nishioka, T. Tsuchiya, W. Namiki, M. Takayanagi, M. Imura, Y. Koide, T. Higuchi, K. Terabe, *Sci. Adv.* **2022,** *8*, eade1156.

[2] J. B. Mallinson, J. K. Steel, Z. E. Heywood, S. J. Studholme, P. J. Bones, S. A. Brown, *Adv. Mater.* **2024,** *36*, 2402319.

[3] T. Shingu, H. Uchiyama, T. Watanabe, Y. Ohno, *Carbon* **2023,** *214*, 118344.

[4] M. Akai-Kasaya, Y. Takeshima, S. Kan, K. Nakajima, T. Oya, T. Asai, *Neuromorph. Comput. Eng.* **2022,** *2*, 014003.

[5] S. Kan, K. Nakajima, T. Asai, M. Akai-Kasaya, *Adv. Sci.* **2022,** *9*, 2104076.

[6] S. Kan, K. Nakajima, Y. Takeshima, T. Asai, Y. Kuwahara, M. Akai-Kasaya, *Phys. Rev. Appl.* **2021,** *15*, 024030.

[7] Z. Liu, Q. Zhang, D. Xie, M. Zhang, X. Li, H. Zhong, G. Li, M. He, D. Shang, C. Wang, L. Gu, G. Yang, K. Jin, C. Ge, *Nat. Commun.* **2023,** *14*, 7176.

[8] X. Liang, Y. Zhong, J. Tang, Z. Liu, P. Yao, K. Sun, Q. Zhang, B. Gao, H. Heidari, H. Qian, H. Wu, *Nat. Commun.* **2022,** *13*, 1549.

[9] Y. Sun, T. Lin, N. Lei, X. Chen, W. Kang, Z. Zhao, D. Wei, C. Chen, S. Pang, L. Hu, L. Yang, E. Dong, L. Zhao, L. Liu, Z. Yuan, A. Ullrich, C. H. Back, J. Zhang, D. Pan, J. Zhao, M. Feng, A. Fert, W. Zhao, *Nat. Commun.* **2023,** *14*, 3434.

[10] Y. Wu, N. T. Duong, Y. C. Chien, S. Liu, K. W. Ang, *Adv. Electron. Mater.* **2023,** *10*, 2300481.

[11] R. Wang, Q. Liang, S. Wang, Y. Cao, X. Ma, H. Wang, Y. Hao, *Appl. Phys. Lett.* **2023,** *123*, 042109.

[12] Y. Zhong, J. Tang, X. Li, B. Gao, H. Qian, H. Wu, *Nat. Commun.* **2021,** *12*, 408.

[13] Z. Chen, W. Li, Z. Fan, S. Dong, Y. Chen, M. Qin, M. Zeng, X. Lu, G. Zhou, X. Gao, J. M. Liu, *Nat. Commun.* **2023,** *14*, 3585.

[14] E. Miranda, G. Milano, C. Ricciardi, *IEEE Trans. Nanotechnol.* **2020,** *19*, 609.

[15] G. Milano, G. Pedretti, K. Montano, S. Ricci, S. Hashemkhani, L. Boarino, D. Ielmini, C. Ricciardi, *Nat. Mater.* **2022,** *21*, 195.

[16] Z. Ma, J. Ge, W. Chen, X. Cao, S. Diao, Z. Liu, S. Pan, *ACS Appl. Mater. Interfaces* **2022,** *14*, 21207.

[17] Z. Ma, J. Ge, W. Chen, X. Cao, S. Diao, H. Huang, Z. Liu, W. Wang, S. Pan, *ACS Appl. Mater. Interfaces* **2022,** *14*, 47941.

[18] Y. Li, L. Loh, S. Li, L. Chen, B. Li, M. Bosman, K.-W. Ang, *Nat. Electron.* **2021,** *4*, 348.

[19] R. Midya, Z. Wang, S. Asapu, X. Zhang, M. Rao, W. Song, Y. Zhuo, N. Upadhyay, Q. Xia, J. J. Yang, *Adv. Intell. Syst.* **2019,** *1*, 1900084.

[20] X. Zhu, Q. Wang, W. D. Lu, *Nat. Commun.* **2020,** *11*, 2439.

[21] C. Du, F. Cai, M. A. Zidan, W. Ma, S. H. Lee, W. D. Lu, *Nat. Commun.* **2017,** *8*, 2204.

[22] K. Liu, B. Dang, T. Zhang, Z. Yang, L. Bao, L. Xu, C. Cheng, R. Huang, Y. Yang, *Adv. Mater.* **2022,** *34*, 2108826.

[23] R. Fang, S. Wang, W. Zhang, K. Ren, W. Sun, F. Wang, J. Lai, P. Zhang, X. Xu, Q. Luo, L. Li, Z. Wang, D. Shang, *Adv. Electron. Mater.* **2024**.

[24] R. Chen, H. Yang, R. Li, G. Yu, Y. Zhang, J. Dong, D. Han, Z. Zhou, P. Huang, L. Liu, X. Liu, J. Kang, *Sci. Adv.* **2024,** *10*, eadl1299.

[25] K. Liu, T. Zhang, B. Dang, L. Bao, L. Xu, C. Cheng, Z. Yang, R. Huang, Y. Yang, *Nat. Electron.* **2022,** *5*, 761.

[26] L. Appeltant, M. C. Soriano, G. Van der Sande, J. Danckaert, S. Massar, J. Dambre, B. Schrauwen, C. R. Mirasso, I. Fischer, *Nat. Commun.* **2011,** *2*, 468.
